# Alcohol consumption, DNA methylation and colorectal cancer risk: Results from pooled cohort studies and Mendelian randomization analysis

**DOI:** 10.1002/ijc.33945

**Published:** 2022-01-31

**Authors:** Xuan Zhou, Lijuan Wang, Jiarui Xiao, Jing Sun, Lili Yu, Han Zhang, Xiangrui Meng, Shuai Yuan, Maria Timofeeva, Philip J. Law, Richard S. Houlston, Kefeng Ding, Malcolm G. Dunlop, Evropi Theodoratou, Xue Li

**Affiliations:** ^1^ Department of Big Data in Health Science School of Public Health, and Center of Clinical Big Data and Analytics of The Second Affiliated Hospital Zhejiang University School of Medicine Hangzhou Zhejiang China; ^2^ College of Public Health Zhengzhou University Zhengzhou Henan China; ^3^ Division of Psychiatry University College of London London UK; ^4^ Unit of Cardiovascular and Nutritional Epidemiology Institute of Environmental Medicine, Karolinska Institutet Stockholm Sweden; ^5^ Danish Institute for Advanced Study (DIAS), Epidemiology, Biostatistics and Biodemography Research Unit, Institute of Public Health, University of Southern Denmark Odense Denmark; ^6^ Cancer Research UK Edinburgh Centre Medical Research Council Institute of Genetics and Cancer, University of Edinburgh Edinburgh UK; ^7^ Division of Genetics and Epidemiology The Institute of Cancer Research London UK; ^8^ Department of Colorectal Surgery and Oncology Key Laboratory of Cancer Prevention and Intervention, Ministry of Education, The Second Affiliated Hospital, Zhejiang University Hangzhou Zhejiang China; ^9^ Centre for Global Health Usher Institute, University of Edinburgh Edinburgh UK

**Keywords:** alcohol, colorectal cancer, DNA methylation, Mendelian randomization

## Abstract

Alcohol consumption is thought to be one of the modifiable risk factors for colorectal cancer (CRC). However, the causality and mechanisms by which alcohol exerts its carcinogenic effect are unclear. We evaluated the association between alcohol consumption and CRC risk by analyzing data from 32 cohort studies and conducted two‐sample Mendelian randomization (MR) analysis to examine for casual relationship. To explore the effect of alcohol related DNA methylation on CRC risk, we performed an epigenetic MR analysis with data from an epigenome‐wide association study (EWAS). We additionally performed gene‐alcohol interaction analysis nested in the UK Biobank to assess effect modification between alcohol consumption and susceptibility genes. We discovered distinct effects of alcohol on CRC incidence and mortality from the meta‐analyses, and genetic predisposition to alcohol drinking was causally associated with an increased CRC risk (OR = 1.79, 95% CI: 1.23‐2.61) using two‐sample MR approaches. In epigenetic MR analysis, two alcohol‐related CpG sites (cg05593667 and cg10045354 mapped to *COLCA1/COLCA2* gene) were identified causally associated with an increased CRC risk (*P* < 8.20 × 10^−4^). Gene‐alcohol interaction analysis revealed that carriage of the risk allele of the eQTL (rs3087967) and mQTL (rs11213823) polymorphism of *COLCA1*/*COLCA2* would interact with alcohol consumption to increase CRC risk (*P*_Interaction_ = .027 and *P*_Interaction_ = .016). Our study provides comprehensive evidence to elucidate the role of alcohol in CRC and highlights that the pathogenic effect of alcohol on CRC could be partly attributed to DNA methylation by regulating the expression of *COLCA1*/*COLCA2* gene.

## Graphical abstract

What's new?

While alcohol consumption is suspected of being a modifiable risk factor for colorectal cancer (CRC), causal relationships and carcinogenic mechanisms linked to alcohol consumption remain unclear. In this study, investigation of interactions between alcohol consumption and susceptibility genes revealed associations between genetic predisposition to alcohol drinking and increased CRC risk. Epigenetic analyses showed that the pathogenic effect of alcohol can be attributed in part to DNA methylation via regulation of the expression of the *COLCA1*/*COLCA2* gene. Polymorphisms in risk alleles in *COLCA1/COLCA2* interacted with alcohol consumption to increase CRC risk, providing insight into how alcohol modulates CRC tumorigenesis.

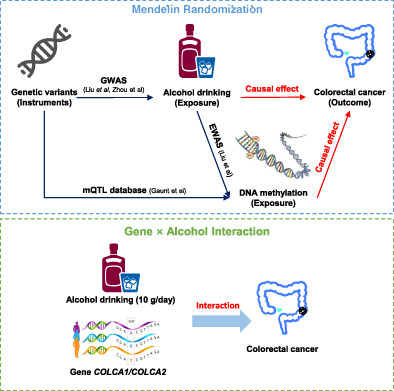

**Abbreviations**
ALSPAC: Avon Longitudinal Study of Parents and Children
ARIES: Accessible Resource for Integrated Epigenomic Studies
AUD: alcohol use disorder
CI: confidence interval
CRC: colorectal cancer
EWAS: epigenome‐wide association study
GSCAN: GWAS and Sequencing Consortium of Alcohol and Nicotine use
GWAS: genome‐wide association study
HR: hazard ratio
IVs: genetic instruments
IVW: inverse variance weighted
mQTLs: methylation quantitative trait loci
MR: Mendelian randomization
MR‐PRESSO: MR pleiotropy residual sum and outlier
OR: odds ratio
PAU: problematic alcohol use
SNPs: single nucleotide polymorphisms

## 1 INTRODUCTION

Colorectal cancer (CRC) is the third most commonly diagnosed cancer worldwide, and the second leading cause of cancer‐related death worldwide, with more than 1.9 million new cases and 935 000 deaths estimated to occur in 2020.^1^ Alcohol drinking is thought to be one of the modifiable risk factors of CRC.^2^ The World Cancer Research Fund and the American Institute for Cancer Research concluded that alcohol intake more than 30 g of ethanol per day (about two drinks a day) is associated with increased risk of CRC,^2^ however, the association between light and moderate levels of alcohol consumption and CRC risk are unclear. A pooled analysis identified a J‐shaped association between alcohol drinking and CRC risk, where moderate alcohol consumption showed a protective effect comparing to nondrinkers,^3^ while other studies reported a positive dose‐response relationship or nonsignificant positive association at moderate levels of alcohol consumption.^4^, ^5^ Additionally, it is uncertain whether the effect of alcohol consumption on cancer risk is different between colon and rectum. One study reported a stronger alcohol‐cancer risk association for the colon,^6^ whereas others discovered a stronger or similar association for the rectum.^7^, ^8^, ^9^ Overall, the observational association between alcohol and CRC risk is inconsistent and these discrepancies could be due to confounding, reverse causation, and other biases inherited from conventional epidemiological studies.

Mendelian randomization (MR) is a method commonly applied in epidemiology to estimate the causal relationship between a modifiable environmental exposure and a medical relevant trait or disease by utilizing genetic variants as instrumental variables (IVs).^10^ Recent meta‐analyses of genome‐wide association studies (GWAS) identified a number of single nucleotide polymorphisms (SNPs) associated with general alcohol consumption (drinks per week),^11^ and the subphenotypes of more severe drinking behaviors and physiological alcohol dependence (alcohol use disorder and problematic alcohol use),^12^ which can be selected as IVs to proxy the genetic predisposition of different alcohol drinking behaviors in MR analyses.

Beyond causality, the underlying mechanisms are not fully understood. Epigenome‐wide association studies (EWAS) showed that alcohol consumption can affect DNA methylation both in blood and tissues,^13^, ^14^ and aberrant patterns of DNA methylation, an important epigenetic mechanism of transcriptional control, can also lead to CRC development.^15^ It has been reported that alcohol drinking could also interact with the genetic polymorphism of several susceptibility genes in the tumorigenesis of CRC.^16^, ^17^ Therefore, we hypothesize that alcohol related DNA methylation and gene‐alcohol interaction might be involved in the pathological effect of alcohol drinking on CRC risk.

In our study, we comprehensively evaluated the relationship between alcohol consumption and CRC risk by performing an updated meta‐analysis of prospective cohort studies and conducting two‐sample Mendelian randomization (MR) analyses for causal inference. To shed light on the underlying biology, we explored the causal effects of alcohol related DNA methylation on the risk of CRC and performed gene‐alcohol interaction analysis nested in the UK Biobank cohort.

## 2 MATERIALS AND METHODS

### 2.1 Meta‐analysis of prospective cohort studies

#### 2.1.1 Literature search

We performed a comprehensive literature search for cohort studies in MEDLINE, EMBASE and BIOSIS citation index, using “alcohol”, “CRC” and “cohort” as the keywords. The detailed search strategy can be seen in Supporting Information Methods. Reference lists of retrieved reviews and meta‐analyses were manually searched to identify additional studies of interest.

Studies were included if they met the following criteria: (i) examined the relationship between alcohol consumption and CRC incidence or mortality; (ii) reported odds ratio (OR), relative risk (RR) or hazard ratio (HR) (or providing sufficient data to compute them) for at least two levels of alcohol consumption vs nondrinkers and/or occasional drinkers (<1 drink/week); (iii) reported standard errors or confidence intervals (CIs) of the risk estimates (or providing sufficient data to calculate them). Studies were excluded if they met any of the following criteria: (i) not prospective cohort studies; (ii) included individuals with prior CRC history; (iii) did not report the independent effect of alcohol on CRC risk; (iv) reported the risk estimates of a specific type of alcoholic beverage only; (v) when more than one study was published for the same cohort, only the most recent and comprehensive one was included and the others were excluded.

### 2.2 Data extraction and quality assessment

The retrieved articles were independently reviewed by two authors to determine the eligibility for inclusion in the meta‐analysis. Doubts and disagreements were resolved by consensus among all the investigators. For each included study, two authors independently extracted the following items: first author, publication year, country, study population, the number (or person‐years) of cases and subjects at risk, age, gender, anatomical subsite, exposure levels and corresponding risk estimates with 95% CIs, outcome, adjustment variables, and duration of follow‐up. As the measurement scale of alcohol was different among included studies, we set grams per day as a standard, considering one drink as 12.5 g, 1 mL as 0.8 g, and 1 oz as 28 g ethanol, unless otherwise specified in original studies.^4^ If alcohol consumption was reported by a range, the midpoint was taken as the exposure dose; and for an open‐ended upper category, the dose was assigned as the lower bound plus three‐quarters of the length of the previous category.^4^, ^18^ We divided alcohol consumption into three categories, considering ≤12.5, ≤50 and >50 g/day as light, moderate and heavy drinking, respectively. When a study reported two or more risk estimates for a single dose category, we used the method developed by Hamling et al to combine them into one single estimate.^19^ The Newcastle‐Ottawa Scale (NOS) was used to evaluate the methodological quality of included studies.^20^ The scale has a score system (ranging from 0 to 9 points) to assess three domains: selection, comparability, and outcome. In the comparability domain, we set age, sex, and BMI as the most important covariates that should be adjusted, and in the outcome section, we regarded more than 5 years follow‐up as enough for outcomes to occur and follow‐up rate larger than 80% as adequacy. We considered the overall score 0‐5, 6‐7, and 8‐9 as low, moderate, and high quality, respectively.

### 2.3 Statistical analysis

We computed a pooled RR for each exposure category compared to nondrinkers and/or occasional drinkers using the inverse variance weighted random‐effects model. Statistical heterogeneity across included studies was evaluated by the *I* ^2^ index. We conducted meta‐analysis for CRC incidence (the risk to develop CRC) and CRC‐specific mortality (the risk of death due to CRC) separately; subgroup analyses for different sex, anatomical site of tumor and study population were also performed. For the dose‐response analysis, we assigned the transformed exposure levels as the doses and their corresponding RR or HR estimates as responses, using cubic‐spline model with 0, 12.5, and 50 g per day alcohol drinking as knots to estimate the dose‐response trend, and the adjusted *R*‐square to quantify the model fit.

### 2.4 Genetic instruments for predisposition to alcohol drinking

We derived genetic instruments for predisposition to general alcohol drinking (drinks per week) from a GWAS conducted by the GWAS and Sequencing Consortium of Alcohol and Nicotine use (GSCAN) in 1.2 million individuals of European ancestry.^11^ Data of drinks per week of all participants were collected using a questionnaire, left‐anchored at 1 and log‐transformed (natural log).^11^ For each SNP that passed quality control, linear regression was applied to calculate the effect sizes of the exposure.^11^ A total of 99 SNPs of genome‐wide significance (*P* < 5 × 10^−8^) were identified to be associated with the phenotype, and 84 SNPs remained after removing those in linkage disequilibrium (*r* ^2^ > .01). To complement with more severe drinking behaviors and alcohol dependence, genetic instruments for alcohol use disorder (AUD) and problematic alcohol use (PAU) were also used. A total of 30 SNPs and 42 SNPs at genome‐wide significance (*P* < 5 × 10^−8^) were identified for AUD and PAU respectively from a GWAS meta‐analysis containing 435 563 individuals of European‐ancestry (Million Veteran Program, Psychiatric Genomics Consortium, and UK Biobank),^12^ and 19 and 26 SNPs remained after clumping those in linkage disequilibrium (*r* ^2^ > .01).

### 2.5 Methylation quantitative trait loci (mQTL) for alcohol‐related DNA methylation

We obtained the DNA methylation pattern related to alcohol consumption from an epigenome‐wide association study with meta‐analysis of 13 population‐based cohorts using whole blood DNA.^21^ Illumina Infinium HumanMethylation450 (HM450) BeadChip was used to measure the level of methylation and the association between alcohol consumption and methylation level of epigenome‐wide CpG sites was adjusted for multiple covariates, such as age, sex, BMI, batch effects and white cell blood counts (ie, CD4 cells, CD8 cells, natural killer cells, B cells and monocytes).^21^ A total of 363 CpG sites were identified to be associated with alcohol consumption at epigenome‐wide significance level (*P*<1 × 10^−7^). Then, we derived genetic variants robustly associated with those CpG sites from an mQTL database based on the Accessible Resource for Integrated Epigenomic Studies (ARIES) project.^22^ The ARIES was launched using the Illumina Infinium HumanMethylation450 (HM450) BeadChip to acquire epigenetic data (CpG sites) and the Illumina Infinium Human Hap550 and 660‐w quad genome‐wide SNP genotyping platform to acquire genetic data (SNPs) on cord blood and peripheral blood samples from 1018 mother‐offspring pairs in the Avon Longitudinal Study of Parents and Children (ALSPAC) cohort.^23^, ^24^, ^25^ The Matrix eQTL software was used for the preliminary association analysis of SNPs with CpG sites.^22^ SNPs at epigenome‐wide association significance (*P* < 1 × 10^−7^) were further analyzed using exact linear regression including covariates in PLINK 1.07, and genome‐wide complex trait analysis (GCTA) was performed to obtain the most representative independent loci in relation to each CpG site.^22^ For each of those CpG sites associated with alcohol consumption, we identified mQTL‐DNA methylation effect estimates at middle‐age time point for the methylation MR analysis.

### 2.6 GWAS summary‐level data of CRC

We investigated the relationship between the selected genetic IVs and CRC risk using summary data from 12 previously reported CRC GWASs. Briefly, these GWASs included individuals of European ancestry from the following studies: CCRR1, CCFR2, COIN, CORSA, Croatia, DACHS, FIN, NSCCG‐OncoArray, SCOT, UK1, VQ58, and Scottish case‐control series, comprising 20 049 cases and 22 661 controls. Comprehensive details of the cases and controls are available in our previously published work.^26^ After standard quality control procedures, a total of 16 871 cases and 26 328 controls were included in the meta‐GWAS analysis.

### 2.7 Two‐sample MR

To test the causality between genetic predisposition to alcohol consumption and CRC risk, drinks per week, AUD, and PAU were considered as the alcohol exposures, and 84, 19, and 26 SNPs were used as the IVs. To investigate the causal effect of alcohol related DNA methylation on the risk of CRC, methylation at each CpG site induced by alcohol was regarded as the exposure and its proxy mQTLs were used as the IVs. For each genetic instrument, *β*_GP_ is the estimate of the genetic association with the exposure (ie, alcohol consumption or DNA methylation) and *β*_GD_ is the estimate of the genetic association with the outcome (ie, CRC). We calculated the effect estimates in CRC risk per SD (SD) increase in alcohol consumption (or DNA methylation) using the formula *β*_GD_/*β*_GP_ (Wald ratio) and combined in a fixed‐effect meta‐analysis after weighing each ratio estimate using the inverse variance weighted (IVW) approach. We additionally undertook multiple analyses to assess the risk of horizontal pleiotropy (a violation of the second MR assumption). First, we used the MR Egger method, which allows for an additional intercept (alpha) term and provides an estimate of directional horizontal pleiotropy.^27^ Then, we used three additional MR methods known to be more robust to the presence of horizontal pleiotropy (but at the expense of statistical power): simple mode, weighted median, and weighted mode.^28^ Given possible instability in MR estimates, we applied the global test, outlier test, and distortion test using the MR pleiotropy residual sum and outlier (MR‐PRESSO) R package as an additional control for pleiotropy.^29^ To control for potential pleiotropic effect on confounders, we queried PhenoScanner^30^ to identify all reported association for the IVs and performed sensitivity analyses by excluding those associated with obesity related traits (ie, BMI, waist circumference, hip circumference, and weight), physical activity, smoking, education, Crohn's disease and inflammatory bowel disease at the threshold of 5 × 10^−8^ in European ancestry samples. MR analyses were performed using the TwoSampleMR R package.^31^

While evaluating the causal effect of DNA methylation at alcohol‐drinking related CpG sites, the effect allele of mQTL was unified to be in the same direction with the effect of alcohol intake on DNA methylation. If there was only one mQTL for a CpG site, only the Wald ratio with its corresponding SE was calculated, but if it was associated with multiple independent mQTLs, the aforementioned IVW approach was applied. Bonferroni correction was applied to account for multiple testing.

### 2.8 Gene‐alcohol interaction analysis nested in the UK Biobank

**FIGURE 1:**
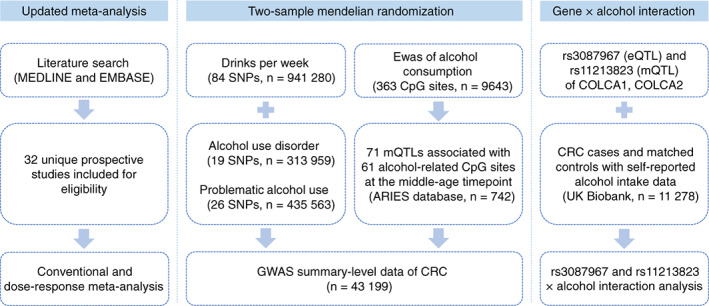
Study design [Color figure can be viewed at wileyonlinelibrary.com]

UK Biobank is a large cohort study that recruited more than half a million people aged 40 to 69 years across the UK during 2006 and 2010. The details of baseline information and biological samples collection can be found elsewhere.^32^ To validate the MR estimates and test whether the effect of alcohol‐related methylation on CRC was distinct by the expression and methylation levels of *COLCA1/COLCA2* gene, we obtained the genotyping data of the eQTL SNP rs3087967^33^ and the mQTL SNP rs11213823 of the *COLCA1*/*COLCA2* gene along with alcohol drinking information and performed gene‐alcohol interaction analysis among incident CRC cases and population‐based controls nested in the UK Biobank. We excluded former drinkers and finally included 1892 incident CRC cases and 9386 matched controls for the analysis after quality control. Various types of alcoholic beverages were transformed into gram per day of ethanol and the amount of consumption was divided into three categories (light, moderate and heavy) as mentioned before. Then we investigated the independent estimates of alcohol drinking (per 10 g/d) using the R function “glm”, the independent estimates of rs3087967 and rs11213823 and the interaction estimates using R function “snp.logistic” in CGEN R package^34^ with two regression models under the framework of a nested case‐control study. We basically adjusted for age and sex of the participants, and additionally adjusted for area deprivation index, red meat consumption, processed meat consumption, aspirin intake, BMI and smoking for the multivariable model. Based on the genotypes of rs3087967 and rs11213823, we further conducted stratification analyses to assess the dose‐response effects of alcohol drinking (per 10 g/d). Time‐to‐event analysis was performed in CRC incident cases to test if the time to CRC occurrence was different among nondrinkers, light, moderate and heavy drinkers stratified by the genotype of rs3087967 and rs11213823. The study design is shown in Figure 1. All analyses were conducted using R version 4.1.0.

## 3 RESULTS

### 3.1 Meta‐analysis of observational studies

A total of 3407 articles were retrieved from MEDLINE, EMBASE and BIOSIS citation index databases. After screening of title, abstract and full text, 32 unique prospective cohort studies were eligible to be included in the meta‐analysis. More details regarding the process of parallel screening can be found in Figure S1. Of them, 25 (78.1%) reported incidence and seven (21.9%) reported mortality as the outcome of interest; 11 (34.4%) were conducted in North America, nine (28.1%) in Europe, 10 (31.3%) in Asia, two (6.3%) in mixed or other areas; five (15.6%) reported estimates for men, two (6.3%) for women, 15 (46.8%) for both men and women in subgroups and 10 (31.2%) for both men and women together; three (9.4%) reported the risk of colon cancer only, 11 (34.4%) reported the risk of colon cancer and rectum cancer separately, and 18 (56.3%) reported the incorporative risk of colorectal cancer; 22 (68.8%) included abstainers whereas eight (25.0%) included occasional drinkers in the reference category (Table S1). The details of all included studies can be seen in Table S2. Quality assessment based on the NOS scale suggested 28 studies (87.5%) as high quality and four (12.5%) as moderate quality (Table S3).

Figure S2 presents the pooled RR estimates for light, moderate and heavy drinking as compared to nondrinkers and occasional drinkers in the risk of CRC incidence and mortality as well as several subgroups stratified by sex, histologic subtypes and main population. No association were observed between light drinking and CRC risk. Significant associations were identified for CRC incidence (RR = 1.24, 95% CI: 1.17‐1.32, *P* = 5.27 × 10^−12^ for moderate drinking, RR = 1.54, 95% CI: 1.15‐2.06, *P* = .004 for heavy drinking) but not for CRC mortality (RR = 0.98, 95% CI: 0.86‐1.12, *P* = .789 for moderate drinking, RR = 1.16, 95% CI: 0.85‐1.57, *P* = .359 for heavy drinking). The association between alcohol drinking and CRC was identified in men (RR = 1.24, 95% CI: 1.15‐1.33, *P* = 7.26 × 10^−9^ for moderate drinking, and RR = 1.45, 95% CI: 1.16‐1.80, *P* = .001 for heavy drinking), while no significant detrimental effect of drinking on CRC risk was observed in women drinkers. Considering colon cancer and rectum cancer separately, moderate and heavy drinking were associated with higher risk (RR = 1.25, 95% CI: 1.14‐1.38, *P* = 5.45 × 10^−6^ for moderate drinking and RR = 1.59, 95% CI: 1.06‐2.38, *P* = .026 for heavy drinking on colon cancer, RR = 1.38, 95% CI: 1.28‐1.48, *P* = 1.65 × 10^−16^ for moderate drinking and RR = 1.92, 95% CI: 1.47‐2.51, *P* = 2.17 × 10^−6^ for heavy drinking on rectum cancer). For different anatomical sites of colon, the risk of distal colon cancer increased both in moderate and heavy drinkers (RR = 1.24, 95% CI: 1.15‐1.35, *P* = 1.25 × 10^−7^ and RR = 1.66, 95% CI: 1.17‐2.36, *P* = .005, respectively) while no significant associations were observed in proximal colon cancer. Focusing on the study population, the risk of CRC increased among moderate and heavy drinkers in European populations as well as Asian populations, while the latter had larger magnitude and wider confidence intervals (RR = 1.20, 95% CI: 1.10‐1.31, *P* = 4.39 × 10^−5^ for moderate drinking and RR = 1.41, 95% CI: 1.16‐1.71, *P* = 4.24 × 10^−4^ for heavy drinking in European populations, RR = 1.29, 95% CI: 1.09‐1.54, *P* = .003 for moderate drinking and RR = 1.53, 95% CI: 1.02‐2.29, *P* = .038 for heavy drinking in Asian populations). In addition, a significant nonlinear dose‐response relationship between alcohol consumption and CRC risk was identified, in which the dose of alcohol was treated as a continuous variable. Results showed that the risk of CRC incidence increased distinctly with increasing dose of alcohol intake between 0 and 50 g per day and increased modestly afterwards (*P* = 1.32 × 10^−7^, adjusted *R*‐square = .18). However, the risk of CRC mortality tended to reveal a J‐shaped association with increasing dose of alcohol intake and the turning point was approximately 25 g per day (*P* = .014, adjusted *R*‐square = .14). Stratification by sex, cancer site and study population showed similar trends as incidence, except for colon cancer that showed an almost linear association (*P* = 6.47 × 10^−11^, adjusted *R*‐square = .45) (Figure S3).

### 3.2 Causal effect of genetic predisposition to alcohol drinking on CRC risk

We firstly calculated the *F*‐statistic of each instrument and no weak instruments were identified (*F*‐statistic > 10, Table S4). Among the instrumental variables for alcohol consumption (drinks per week, AUD and PAU), 84 (100%), 18 (94.7%) and 24 (92.3%) were matched with GWAS summary‐level data of CRC, respectively. Using the IVW approach, we observed a significant causal association between genetically predicted drinks per week and increased risk of CRC: for per one SD increase of log‐transformed alcoholic drinks per week, the risk of CRC would increase 79% (OR = 1.79, 95% CI: 1.23‐2.61, *P* = .003). Genetic predisposition to PAU was nominally associated with increased risk of CRC (OR = 1.53, 95% CI: 1.02‐2.29, *P* = .040), while no significant causal effect was observed between genetic predisposition to AUD and CRC risk (OR = 1.33, 95% CI: 0.95‐1.85, *P* = .093) (Table 1), which might be due to the limited statistical power. There was substantial heterogeneity in all MR analyses except for that of AUD, but no apparent horizontal pleiotropy was observed in MR‐Egger regression (*P*_Intercept_ = .717 for drinks per week, *P*_Intercept_ = .174 for AUD, *P*_Intercept_ = .093 for PAU). Using MR‐PRESSO approach, we discovered one outlier among the IVs for drinks per week, one outlier among the IVs for PAU but no outlier among the IVs for AUD (Table S5); the evidence of causal association remained for drinks per week (OR = 1.93, 95% CI: 1.36‐2.73, *P* = 3.90 × 10^−4^), but the evidence against the null hypothesis of no association was limited for PAU (OR = 1.40, 95% CI: 0.99‐1.98, *P* = .071) after removing the outlier. As sensitivity analyses, we excluded IVs associated with potential confounders (Table S6) and observed evidence for the causal effect of drinks per week on CRC risk (OR = 1.77, 95% CI: 1.17‐2.29, *P* = .007), but limited evidence for the effect of AUD and PAU on CRC risk due to the inadequate study power (OR = 1.31, 95% CI: 0.86‐1.98, *P* = .206 for AUD and OR = 1.27, 95% CI: 0.88‐1.81, *P* = .199 for PAU).

**TABLE 1:** Two‐sample Mendelian randomization estimates for the relationship between alcohol consumption and colorectal cancer risk

Exposure	SNPs	MR method	OR (95% CI)	*P*_Effect_	*P*_Heterogeneity_	*P*_Intercept_
Main analysis						
Drinks per week	84	IVW	1.79 (1.23, 2.61)	.003	1.39 × 10^−4^	
		MR Egger	2.03 (0.93, 4.43)	.080	1.10 × 10^−4^	.717
		Weighted median	1.49 (0.91, 2.43)	.112		
		Simple mode	1.55 (0.44, 5.50)	.498		
		Weighted mode	1.19 (0.59, 2.41)	.635		
		MR‐PRESSO	1.93 (1.36, 2.73)	3.90 × 10^−4^		
Alcohol use disorder	18	IVW	1.33 (0.95, 1.85)	.093	.110	
		MR Egger	0.90 (0.49, 1.68)	.753	.155	.174
		Weighted median	1.13 (0.76, 1.69)	.541		
		Simple mode	1.62 (0.72, 3.65)	.257		
		Weighted mode	1.04 (0.65, 1.65)	.878		
		MR‐PRESSO	1.33 (0.95, 1.85)	.111		
Problematic alcohol use	24	IVW	1.53 (1.02, 2.29)	.040	.002	
		MR Egger	0.91 (0.45, 1.83)	.787	.006	.093
		Weighted median	1.07 (0.69, 1.65)	.759		
		Simple mode	0.86 (0.35, 2.09)	.736		
		Weighted mode	0.95 (0.60, 1.50)	.819		
		MR‐PRESSO	1.40 (0.99, 1.98)	.071		
Sensitivity analysis						
Drinks per week	63	IVW	1.77 (1.17, 2.69)	.007	.005	
		MR Egger	1.66 (0.75, 3.66)	.216	.004	.846
		Weighted median	1.53 (0.90, 2.60)	.114		
		Simple mode	2.25 (0.58, 8.69)	.243		
		Weighted mode	1.30 (0.63, 2.68)	.472		
		MR‐PRESSO	1.96 (1.36, 2.83)	6.38 × 10^−4^		
Alcohol use disorder	13	IVW	1.31 (0.86, 1.98)	.206	.063	
		MR Egger	0.95 (0.46, 1.98)	.901	.072	.325
		Weighted median	1.15 (0.74, 1.78)	.546		
		Simple mode	1.43 (0.57, 3.56)	.461		
		Weighted mode	1.06 (0.64, 1.76)	.827		
		MR‐PRESSO	1.31 (0.86, 1.98)	.230		
Problematic alcohol use	17	IVW	1.27 (0.88, 1.81)	.199	.271	
		MR Egger	0.99 (0.55, 1.79)	.972	.279	.321
		Weighted median	1.08 (0.69, 1.69)	.745		
		Simple mode	0.87 (0.33, 2.30)	.776		
		Weighted mode	1.01 (0.63, 1.64)	.955		
		MR‐PRESSO	1.27 (0.88, 1.81)	.218		

### 3.3 Causal effect of alcohol‐related DNA methylation on CRC risk

We identified 363 CpG sites correlated to alcohol consumption of European ancestry from the EWAS conducted by Liu et al.^21^ A total of 71 mQTLs were identified to be robustly associated with 61 CpG sites from ARIES database at the timepoint of middle age using GCTA results and included in the methylation MR analysis, among which 70 (98.6%) mQTLs were cis mQTLs (Table S7). To assess the causal effect of DNA methylation at alcohol consumption‐related CpG sites on CRC risk, we conducted two‐sample MR analyses using the identified mQTLs in the CRC GWAS summary data (Table S8). Eight CpG sites (cg03575969, cg05593667, cg10045354, cg10456541, cg12662084, cg17390562, cg22871253 and cg26312998) were identified to be causally associated with CRC risk at nominal significance level (*P* < .05) and two of them (cg05593667, cg10045354) survived Bonferroni correction (*P* < .05/61 = 8.20 × 10^−4^) (Table 2). CpG site cg10045354 was significantly associated with an increased risk of CRC (OR = 1.67, 95% CI: 1.47‐1.89, *P* = 3.54 × 10^−16^), and located on the CpG island mapping to gene *COLCA2* and *COLCA1*, which are key molecules involved in immunity and defense and have been identified as CRC susceptibility genes (Figure S4). To investigate whether the mQTL rs11213823 of cg10045354 would influence the expression of gene *COLCA2* and *COLCA1* in colon tissue, we queried on GTEx Portal and found it was a strong eQTL of gene *COLCA2* (Figures S5 and S6). For the other identified CpG site cg05593667 which showed a casual association with CRC (OR = 0.89, 95% CI: 0.84‐0.95, *P* = 1.25 × 10^−4^), we did not find any mapped gene(s) regulated by this CpG site and little is known about its nearest gene.

**TABLE 2:** Two‐sample Mendelian randomization estimates for alcohol‐related CpG sites and colorectal cancer risk

CpG sites	Chr	Position	Nearest gene (s)	Method	SNP	OR (95% CI)	*P* value
**cg10045354**	**11**	**111 169 427**	***COLCA2, COLCA1***	**Wald ratio**	**1**	**1.67 (1.47, 1.89)**	**3.54 × 10** ^**−16**^
**cg05593667**	**6**	**35 490 744**	–	**Wald ratio**	**1**	**0.89 (0.84, 0.95)**	**1.25 × 10** ^**−4**^
cg03575969	10	82 172 508	*STARD10, ARAP1*	Wald ratio	1	1.17 (1.05, 1.31)	5.91 × 10^−3^
cg22871253	6	159 238 744	*EZR*	Wald ratio	1	1.12 (1.02, 1.24)	2.08 × 10^−2^
cg12662084	6	17 809 126	*KIF13A*	Wald ratio	1	0.91 (0.84, 0.99)	2.31 × 10^−2^
cg26312998	6	43 337 775	*ZNF318*	Wald ratio	1	1.05 (1.01, 1.10)	2.80 × 10^−2^
cg17390562	6	159 238 463	*EZR*	Wald ratio	1	1.10 (1.01, 1.19)	3.48 × 10^−2^
cg10456541	2	8 721 512	–	Wald ratio	1	1.14 (1.01, 1.30)	3.94 × 10^−2^

*Note*: The bold ones were those that survived multiple‐testing correction (Bonferroni *P* < .05).

Abbreviation: Chr, chromosome.

### 3.4 Gene‐alcohol interaction effect on the risk of CRC

**FIGURE 2:**
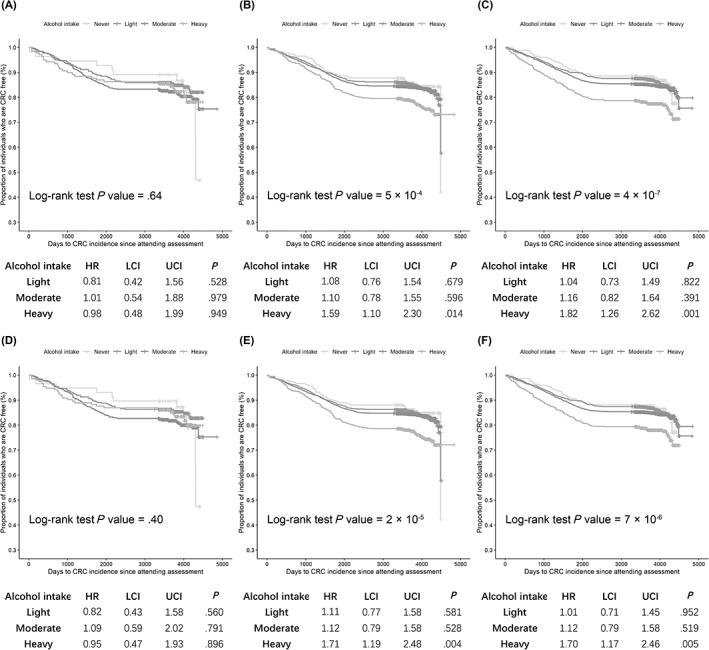
Effect of alcohol drinking on the risk of CRC stratified by the genotype of rs3087967 and rs11213823 in the UK Biobank: (A) rs3087967 CC genotype; (B) rs3087967 CT genotype; (C) rs3087967 TT genotype; (D) rs11213823 CC genotype; (E) rs11213823 CT genotype; (F) rs11213823 TT genotype [Color figure can be viewed at wileyonlinelibrary.com]

The baseline characteristics of the incident CRC cases and controls included for gene‐alcohol interaction analysis were summarized in Table S9. Table 3 shows the effect estimates of alcohol drinking (per 10 g/day), rs3087967, rs11213823 and their interaction effects on the risk of CRC. When considering alcohol drinking and the two genetic variants independently, we observed that 10 g alcohol intake per day was significantly associated with 6% higher risk of CRC (OR = 1.06, 95% CI: 1.04‐1.09, *P* = 3.86 × 10^−8^), rs3087967 was correlated with 8% higher risk of CRC (OR = 1.08, 95% CI: 1.01‐1.16, *P* = .018) and rs11213823 was correlated with 7% higher risk of CRC (OR = 1.08, 95% CI: 1.00‐1.15, *P* = .035) after adjustment for a wide range of covariates. When examining the gene‐alcohol interaction effect, we found evidence for rs3087967‐alcohol and rs11213823‐alcohol interaction on CRC risk (*P*_Interaction_ = .027 and *P*_Interaction_ = .016, respectively). When assessing the dose‐response effect of alcohol drinking (per 10 g/d) stratified by the genotypes of rs3087967 and rs11213823, no significant association was observed among those who carried no risk allele of rs3087967 (OR = 1.01, 95% CI: 0.95‐1.09, *P* = .710) and rs11213823 (OR = 1.01, 95% CI: 0.94‐1.08, *P* = .822). However, alcohol intake was significantly linked with 7% and 8% higher risk of CRC among those with rs3087967 CT and TT genotypes (OR = 1.07, 95% CI: 1.04‐1.11, *P* = 1.54 × 10^−5^ and OR = 1.08, 95% CI: 1.05‐1.12, *P* = 4.43 × 10^−7^), and 6% and 7% higher risk of CRC among those with rs11213823 CT and TT genotypes (OR = 1.06, 95% CI: 1.03‐1.10, *P* = 1.72 × 10^−4^ and OR = 1.07, 95% CI: 1.04‐1.11, *P* = 1.58 × 10^−5^) for 10 g alcohol intake per day (Table 4). In the time‐to‐event multivariable Cox regression models, we observed significant higher risk of CRC development among heavy drinkers, with the HR of 1.59 (95% CI: 1.10‐2.30, *P* = .014) and 1.82 (95% CI: 1.26‐2.62, *P* = .001) for carriers of rs3087967 CT and TT genotypes, and the HR of 1.71 (95% CI: 1.19‐2.48, *P* = .004) and 1.70 (95% CI: 1.17‐2.46, *P* = .005) for carriers of rs11213823 CT and TT genotypes comparing to never drinkers (Figure 2).

**TABLE 3:** Gene‐alcohol interaction estimates for the risk of CRC nested in the UK Biobank

Variables	Basic model		Multivariable model	
	OR (95% CI)	*P* value	OR (95% CI)	*P* value
Alcohol (10 g/day)	1.08 (1.05, 1.10)	4.10 × 10^−12^	1.06 (1.04, 1.09)	3.86 × 10^−8^
rs3087967	1.08 (1.01, 1.15)	.022	1.08 (1.01, 1.16)	.018
rs11213823	1.07 (1.00, 1.14)	.043	1.07 (1.00, 1.15)	.035
rs3087967 × Alcohol (10 g/day)	1.04 (1.01, 1.06)	.001	1.03 (1.00, 1.05)	.027
rs11213823 × Alcohol (10 g/day)	1.04 (1.02, 1.06)	.001	1.03 (1.00, 1.05)	.016

*Note*: Basic model: adjusted for age and sex; multivariable model: additionally adjusted for area deprivation index, red meat consumption, processed meat consumption, aspirin intake, BMI and smoking.

**TABLE 4:** Dose‐response effect of alcohol drinking on the risk of CRC stratified by the genotype of rs3087967 in the UK Biobank

Alcohol (10 g/day)	Basic model		Multivariable model	
	OR (95% CI)	*P* value	OR (95% CI)	*P* value
rs3087967 CC	1.03 (0.97, 1.10)	.392	1.01 (0.95, 1.09)	.710
rs3087967 CT	1.07 (1.04, 1.11)	6.99 × 10^−6^	1.07 (1.04, 1.11)	1.54 × 10^−5^
rs3087967 TT	1.09 (1.06, 1.13)	7.09 × 10^−9^	1.08 (1.05, 1.12)	4.43 × 10^−7^
rs11213823 CC	1.03 (0.96, 1.09)	.401	1.01 (0.94, 1.08)	.822
rs11213823 CT	1.07 (1.04, 1.11)	5.66 × 10^−6^	1.06 (1.03, 1.10)	1.72 × 10^−4^
rs11213823 TT	1.09 (1.06, 1.12)	5.88 × 10^−8^	1.07 (1.04, 1.11)	1.58 × 10^−5^

*Note*: Basic model: adjusted for age and sex; multivariable model: additionally adjusted for area deprivation index, red meat consumption, processed meat consumption, aspirin intake, BMI and smoking.

## 4 DISCUSSION

In our study, we conducted an updated meta‐analysis of prospective cohort studies to explore the observational association between alcohol consumption and CRC risk, and then evaluated the causal impact of alcohol drinking on CRC risk using two‐sample MR approaches. We additionally performed epigenetic MR analysis from the perspective of DNA methylation and gene‐alcohol interaction analysis nested in the UK Biobank to understand how alcohol consumption modulates CRC risk.

The updated meta‐analysis revealed that moderate and heavy drinking were observationally associated with an increased risk of CRC incidence but not for mortality. Dose‐response analysis also illustrated distinct dose‐risk manners: the risk of CRC incidence increased distinctly when alcohol consumption ranged from 0 to 50 g per day and modestly afterwards, nonetheless, the risk of CRC morality showed a J‐shaped association, with the turning point at approximately 25 g per day. Taken these together, it is possible that the pathogenic effect of alcohol differs in terms of CRC incidence and mortality, but due to the limited adjusted *R*‐square, further studies are warranted to proof our findings. Although we confined the meta‐analysis to include only prospective cohort studies, these results should be interpreted with caution due to potential reverse causation, for that only eight studies set a lag time between the assessment of alcohol consumption and CRC occurrence. Using genetic instruments from two large‐scale GWASs in European ancestry as proxies,^11^, ^12^ we appraised the causal relationship between genetic predisposition to alcohol consumption and the risk of CRC through two‐sample MR analyses, in which the phenotype of “drinks per week” represents the general exposure of alcohol consumption, and other two phenotypes “AUD” and “PAU” reflects pathological drinking behaviors. Convincing evidence in support of a significant causal effect of drinks per week and suggestive evidence for a nominally significant causal effect of PAU with CRC were observed. These findings are similar to a previously published meta‐analysis based on a MR approach by Wang et al who used the *aldehyde dehydrogenase 2* (*ALDH2*) variant as a surrogate of alcohol exposure and reported an odds ratio of 1.31 (95% CI: 1.01‐1.70) supporting the role of alcohol in colorectal carcinogenesis.^35^

Along with observational association, the mechanisms by which alcohol consumption exerts its carcinogenic effect are not fully understood. One possible hypothesis is that DNA methylation may mediate the effects of alcohol on CRC risk. Alcohol may influence DNA methylation patterns by inhibiting the activity and expression of enzymes involved in DNA methylation, such as DNA methyltransferases.^36^, ^37^, ^38^, ^39^ To appraise the causal effect of DNA methylation induced by alcohol consumption on CRC risk, we conducted a two‐sample MR using mQTLs of alcohol drinking related CpG sites as proxies. We found two CpG sites (cg05593667, cg10045354) significantly associated with CRC risk after multiple correction. The CpG site cg10045354 was annotated to genes *COLCA1* and *COLCA2*, which have been identified as genes whose expression levels were significantly associated with CRC susceptibility as reported by previous GWAS.^40^

A number of studies have examined the interactions between alcohol intake and CRC susceptibility genes to investigate any effect modification. For instance, Gong et al identified interactions between alcohol consumption and variants in the 9q22.32/*HIATL1* region, and found that light to moderate drinkers with rs9409565 CT and TT genotype were associated with a lower risk of CRC compared to nondrinkers or occasional drinkers.^16^ Our previous study reported a weak evidence suggesting that heavy drinkers with G allele of MTR A2756G variant conferred higher risk of CRC.^41^ To validate our MR findings and test whether the effect of alcohol‐related methylation on CRC was influenced by the expression level of COLCA1/COLCA2 gene, we performed alcohol‐gene interaction analysis using the eQTL rs3087967 and mQTL rs11213823. Our study identified novel alcohol‐gene interactions at both the eQTL and mQTL of *COLCA1*/*COLCA2* in terms of CRC risk. Dose‐response analysis stratified by the genotypes provided supporting evidence for the dose dependent carcinogenesis effect of alcohol drinking on CRC risk among risk allele carriers of rs3087967 and rs11213823, and time‐to‐event analysis revealed that the effect was more prominent among heavy drinkers when comparing with light and moderate drinkers. Our findings yielded insight to understand how alcohol interacts with the methylation and expression level of *COLCA1*/*COLCA2* gene to modulate the tumorigenesis of CRC.

*COLCA1* is a protein‐coding gene and expressed in many cells that are frequently involved in immunity and defense, such as eosinophils and mast cells.^42^ Eosinophils can induce apoptosis and kill tumor cells via releasing ECP, EDN, TNF and granzyme,^43^ while mast cells may be damaging to tumor cells through cytokines and proteolytic enzyme secretion,^44^ indicating that *COLCA1* may exert its tumoricidal functions through immunity. *COLCA2* is found to be coregulated with *COLCA1* and is present in many epithelial cells. The expression of *COLCA2* is increased in immune and other cells of the microenvironment and reduced in tumor cells,^45^ which suggests a potential protective role of *COLCA2* in antitumor immunity. Alcohol intake may exert the carcinogenic effect on CRC by downregulating the expression of *COLCA1/COLCA2* gene through epigenetic modification.

Our study has several strengths, which include the comprehensive evaluations of the association between alcohol consumption with CRC outcomes through updated meta‐analysis and two‐sample MR analyses. Furthermore, by integrating several large‐scale datasets, causal effects of alcohol related CpG sites on CRC risk and positive interaction effect between susceptibility gene and alcohol drinking were detected to possibly explain potential pathogenic mechanisms underlying significant observations. However, our study also has limitations. Our MR findings were concluded within the European descents, while a strong genetic determinant of alcohol consumption on *aldehyde dehydrogenase 2* (*ALDH2* rs671) which is East Asian‐specific genetic polymorphism^46^ was not investigated in our study. The mQTL effects used in the methylation MR analysis were obtained from the ARIES middle‐age time point, which only contained females at this time point. Without access to the full EWAS summary‐level data, we were not able to perform mediation analysis to estimate the magnitude of carcinogenesis effect of alcohol drinking on CRC mediated by DNA methylation. To better control the potential covariates, we performed the gene‐alcohol interaction under the framework of a nested case‐control study in UK biobank, further study is needed to validate the findings in a perspective way.

In conclusion, our study found that genetic predisposition to alcohol drinking was causally associated with increased CRC risk and the pathogenic effect of alcohol could be partly attributed to DNA methylation via regulating the expression of *COLCA1*/*COLCA2* gene; the eQTL rs3087967 and mQTL rs11213823 polymorphism of *COLCA1*/*COLCA2* gene would interact with alcohol consumption to increase the risk of CRC. Further studies with larger‐scale and tissue‐specific dataset are warranted to confirm the findings by providing molecular evidence.

## CONFLICT OF INTEREST

The authors declare no potential conflict of interests.

## ETHICS STATEMENT

All studies were approved by the respective institutional review boards and conducted with appropriate ethical criteria in each country and in accordance with the Declaration of Helsinki.

## Supporting information


**Appendix S1**: Supporting Information

## Data Availability

The results of our study are included in this published article and its Supporting Information files. The UK Biobank is an open access resource and bona fide researchers can apply to use the UK Biobank dataset by registering and applying at http://ukbiobank.ac.uk/register-apply/. Further information is available from the corresponding author upon request.

## References

[1] Sung H , Ferlay J , Siegel RL , et al. Global cancer statistics 2020: GLOBOCAN estimates of incidence and mortality worldwide for 36 cancers in 185 countries. CA Cancer J Clin. 2021;71:209‐249. 33538338 10.3322/caac.21660

[2] World Cancer Research Fund/American Institute for Cancer Research Diet, Nutrition, Physical Activity and Colorectal Cancer. Continuous Update Project Expert Report; 2018. http://dietandcancerreport.org. Accessed 6 August 2021.

[3] McNabb S , Harrison TA , Albanes D , et al. Meta‐analysis of 16 studies of the association of alcohol with colorectal cancer. Int J Cancer. 2020;146:861‐873. 31037736 10.1002/ijc.32377 PMC6819207

[4] Bagnardi V , Rota M , Botteri E , et al. Alcohol consumption and site‐specific cancer risk: a comprehensive dose‐response meta‐analysis. Br J Cancer. 2015;112:580‐593. 25422909 10.1038/bjc.2014.579 PMC4453639

[5] Klarich DS , Brasser SM , Hong MY . Moderate alcohol consumption and colorectal cancer risk. Alcohol Clin Exp Res. 2015;39:1280‐1291. 26110674 10.1111/acer.12778

[6] Sarich P , Canfell K , Egger S , et al. Alcohol consumption, drinking patterns and cancer incidence in an Australian cohort of 226,162 participants aged 45 years and over. Br J Cancer. 2021;124:513‐523. 33041337 10.1038/s41416-020-01101-2 PMC7853127

[7] Burón Pust A , Alison R , Blanks R , et al. Heterogeneity of colorectal cancer risk by tumour characteristics: large prospective study of UK women. Int J Cancer. 2017;140:1082‐1090. 27859268 10.1002/ijc.30527 PMC5347941

[8] Murphy N , Ward HA , Jenab M , et al. Heterogeneity of colorectal cancer risk factors by anatomical subsite in 10 European countries: a multinational cohort study. Clin Gastroenterol Hepatol. 2019;17:1323‐31.e6. 30056182 10.1016/j.cgh.2018.07.030 PMC6542674

[9] Park SY , Wilkens LR , Setiawan VW , Monroe KR , Haiman CA , Le Marchand L . Alcohol intake and colorectal cancer risk in the multiethnic cohort study. Am J Epidemiol. 2019;188:67‐76. 30239578 10.1093/aje/kwy208 PMC6321802

[10] Davey Smith G , Hemani G . Mendelian randomization: genetic anchors for causal inference in epidemiological studies. Hum Mol Genet. 2014;23:R89‐R98. 25064373 10.1093/hmg/ddu328 PMC4170722

[11] Liu M , Jiang Y , Wedow R , et al. Association studies of up to 1.2 million individuals yield new insights into the genetic etiology of tobacco and alcohol use. Nat Genet. 2019;51:237‐244. 30643251 10.1038/s41588-018-0307-5 PMC6358542

[12] Zhou H , Sealock JM , Sanchez‐Roige S , et al. Genome‐wide meta‐analysis of problematic alcohol use in 435,563 individuals yields insights into biology and relationships with other traits. Nat Neurosci. 2020;23:809‐818. 32451486 10.1038/s41593-020-0643-5 PMC7485556

[13] Klutstein M , Nejman D , Greenfield R , Cedar H . DNA methylation in cancer and aging. Cancer Res. 2016;76:3446‐3450. 27256564 10.1158/0008-5472.CAN-15-3278

[14] Wilson LE , Xu Z , Harlid S , et al. Alcohol and DNA methylation: an epigenome‐wide association study in blood and normal breast tissue. Am J Epidemiol. 2019;188:1055‐1065. 30938765 10.1093/aje/kwz032 PMC6545285

[15] Tse JWT , Jenkins LJ , Chionh F , Mariadason JM . Aberrant DNA methylation in colorectal cancer: what should we target? Trends Cancer. 2017;3:698‐712. 28958388 10.1016/j.trecan.2017.08.003

[16] Gong J , Hutter CM , Newcomb PA , et al. Genome‐wide interaction analyses between genetic variants and alcohol consumption and smoking for risk of colorectal cancer. PLoS Genet. 2016;12:e1006296. 27723779 10.1371/journal.pgen.1006296 PMC5065124

[17] Iwasaki M , Budhathoki S , Yamaji T , et al. Inclusion of a gene‐environment interaction between alcohol consumption and the aldehyde dehydrogenase 2 genotype in a risk prediction model for upper aerodigestive tract cancer in Japanese men. Cancer Sci. 2020;111:3835‐3844. 32662535 10.1111/cas.14573 PMC7540993

[18] Patra J , Bakker R , Irving H , Jaddoe VWV , Malini S , Rehm J . Dose‐response relationship between alcohol consumption before and during pregnancy and the risks of low birthweight, preterm birth and small for gestational age (SGA)—a systematic review and meta‐analyses. BJOG. 2011;118:1411‐1421. 21729235 10.1111/j.1471-0528.2011.03050.x PMC3394156

[19] Hamling J , Lee P , Weitkunat R , Ambühl M . Facilitating meta‐analyses by deriving relative effect and precision estimates for alternative comparisons from a set of estimates presented by exposure level or disease category. Stat Med. 2008;27:954‐970. 17676579 10.1002/sim.3013

[20] Wells GA, Shea B, O'Connell D, et al. The Newcastle‐Ottawa Scale (NOS) for assessing the quality of nonrandomised studies in meta‐analyses.

[21] Liu C , Marioni RE , Hedman AK , et al. A DNA methylation biomarker of alcohol consumption. Mol Psychiatry. 2018;23:422‐433. 27843151 10.1038/mp.2016.192 PMC5575985

[22] Gaunt TR , Shihab HA , Hemani G , et al. Systematic identification of genetic influences on methylation across the human life course. Genome Biol. 2016;17:61. 27036880 10.1186/s13059-016-0926-z PMC4818469

[23] Boyd A , Golding J , Macleod J , et al. Cohort profile: the ‘children of the 90s’—the index offspring of the Avon longitudinal study of parents and children. Int J Epidemiol. 2013;42:111‐127. 22507743 10.1093/ije/dys064 PMC3600618

[24] Fraser A , Macdonald‐Wallis C , Tilling K , et al. Cohort profile: the Avon longitudinal study of parents and children: ALSPAC mothers cohort. Int J Epidemiol. 2013;42:97‐110. 22507742 10.1093/ije/dys066 PMC3600619

[25] Relton CL , Gaunt T , McArdle W , et al. Data resource profile: accessible resource for integrated epigenomic studies (ARIES). Int J Epidemiol. 2015;44:1181‐1190. 25991711 10.1093/ije/dyv072 PMC5593097

[26] Li X , Timofeeva M , Spiliopoulou A , et al. Prediction of colorectal cancer risk based on profiling with common genetic variants. Int J Cancer. 2020;147:3431‐3437. 32638365 10.1002/ijc.33191

[27] Bowden J , Davey Smith G , Burgess S . Mendelian randomization with invalid instruments: effect estimation and bias detection through Egger regression. Int J Epidemiol. 2015;44:512‐525. 26050253 10.1093/ije/dyv080 PMC4469799

[28] Burgess S , Scott RA , Timpson NJ , Davey Smith G , Thompson SG . Using published data in Mendelian randomization: a blueprint for efficient identification of causal risk factors. Eur J Epidemiol. 2015;30:543‐552. 25773750 10.1007/s10654-015-0011-z PMC4516908

[29] Verbanck M , Chen CY , Neale B , Do R . Detection of widespread horizontal pleiotropy in causal relationships inferred from Mendelian randomization between complex traits and diseases. Nat Genet. 2018;50:693‐698. 29686387 10.1038/s41588-018-0099-7 PMC6083837

[30] Staley JR , Blackshaw J , Kamat MA , et al. PhenoScanner: a database of human genotype‐phenotype associations. Bioinformatics. 2016;32:3207‐3209. 27318201 10.1093/bioinformatics/btw373 PMC5048068

[31] Hemani G , Zheng J , Elsworth B , et al. The MR‐base platform supports systematic causal inference across the human phenome. Elife. 2018;7:1‐29. 10.7554/eLife.34408 PMC5976434 29846171

[32] Bycroft C , Freeman C , Petkova D , et al. The UK biobank resource with deep phenotyping and genomic data. Nature. 2018;562:203‐209. 30305743 10.1038/s41586-018-0579-z PMC6786975

[33] Vaughan‐Shaw PG , Timofeeva M , Ooi LY , et al. Differential genetic influences over colorectal cancer risk and gene expression in large bowel mucosa. Int J Cancer. 2021;149:1100‐1108. 33937989 10.1002/ijc.33616

[34] Song M , Wheeler W , Caporaso NE , Landi MT , Chatterjee N . Using imputed genotype data in the joint score tests for genetic association and gene‐environment interactions in case‐control studies. Genet Epidemiol. 2018;42:146‐155. 29178451 10.1002/gepi.22093 PMC5811375

[35] Wang J , Wang H , Chen Y , Hao P , Zhang Y . Alcohol ingestion and colorectal neoplasia: a meta‐analysis based on a Mendelian randomization approach. Colorectal Dis. 2011;13:e71‐e78. 21114754 10.1111/j.1463-1318.2010.02530.x

[36] Liu X , Liu L , Dong Z , et al. Expression patterns and prognostic value of m(6)A‐related genes in colorectal cancer. Am J Transl Res. 2019;11:3972‐3991. 31396313 PMC6684930

[37] Yang X , Zhang S , He C , et al. METTL14 suppresses proliferation and metastasis of colorectal cancer by down‐regulating oncogenic long non‐coding RNA XIST. Mol Cancer. 2020;19:46. 32111213 10.1186/s12943-020-1146-4 PMC7047419

[38] Yue C , Chen J , Li Z , Li L , Chen J , Guo Y . microRNA‐96 promotes occurrence and progression of colorectal cancer via regulation of the AMPKα2‐FTO‐m6A/MYC axis. J Exp Clin Cancer Res. 2020;39:240. 33183350 10.1186/s13046-020-01731-7 PMC7659164

[39] Chen H , Gao S , Liu W , et al. RNA N(6)‐methyladenosine methyltransferase METTL3 facilitates colorectal cancer by activating the m(6)A‐GLUT1‐mTORC1 axis and is a therapeutic target. Gastroenterology. 2021;160:1284‐1300. 33217448 10.1053/j.gastro.2020.11.013

[40] Peltekova VD , Lemire M , Qazi AM , et al. Identification of genes expressed by immune cells of the colon that are regulated by colorectal cancer‐associated variants. Int J Cancer. 2014;134:2330‐2341. 24154973 10.1002/ijc.28557 PMC3949167

[41] Theodoratou E , Timofeeva M , Li X , Meng X , Ioannidis JPA . Nature, nurture, and cancer risks: genetic and nutritional contributions to cancer. Annu Rev Nutr. 2017;37:293‐320. 28826375 10.1146/annurev-nutr-071715-051004 PMC6143166

[42] Vesely MD , Kershaw MH , Schreiber RD , Smyth MJ . Natural innate and adaptive immunity to cancer. Annu Rev Immunol. 2011;29:235‐271. 21219185 10.1146/annurev-immunol-031210-101324

[43] Legrand F , Driss V , Delbeke M , et al. Human eosinophils exert TNF‐α and granzyme A‐mediated tumoricidal activity toward colon carcinoma cells. J Immunol. 2010;185:7443‐7451. 21068403 10.4049/jimmunol.1000446

[44] Mukai K , Tsai M , Saito H , Galli SJ . Mast cells as sources of cytokines, chemokines, and growth factors. Immunol Rev. 2018;282:121‐150. 29431212 10.1111/imr.12634 PMC5813811

[45] Closa A , Cordero D , Sanz‐Pamplona R , et al. Identification of candidate susceptibility genes for colorectal cancer through eQTL analysis. Carcinogenesis. 2014;35:2039‐2046. 24760461 10.1093/carcin/bgu092 PMC4146415

[46] Koyanagi YN , Nakatochi M , Ito H , et al. Genotype‐stratified GWAS meta‐analysis reveals novel loci associated with alcohol consumption. medRxiv. 2021. doi:10.1101/2021.06.02.21258094

