# Supplementary material for: Alcohol consumption, DNA methylation and colorectal cancer risk: Results from pooled cohort studies and Mendelian randomization analysis
Source: Int J Cancer. 2022 Jan 31;151(1):83–94. doi: 10.1002/ijc.33945 (PMC9487984; doi:10.1002/ijc.33945)
Supplement: Supplementary file 1 — Appendix S1: Supporting Information [file IJC-151-83-s001.pdf]

Alcohol consumption, DNA methylation and colorectal cancer risk: results from pooled cohort studies and Mendelian randomisation analysis

Xuan Zhou[1†], Lijuan Wang[1†], Jiarui Xiao[1†], Jing Sun[1], Lili Yu[1], Han Zhang[2], Xiangrui Meng[3], Shuai Yuan[4], Maria Timofeeva[5,6], Philip J Law[7], Richard S Houlston[7], Kefeng Ding[8], Malcolm Dunlop[6], Evropi Theodoratou[6,9*], Xue Li[1*]

Contents

Supplementary materials & methods 2

Detailed search strategy 2

Two-sample MR 3

Supplementary tables 5

Table S1. Summarized characteristics of included studies. 5

Table S2. Detailed characteristics of included studies. 6

Table S3. Quality assessment of included studies using the Newcastle-Ottawa Scale. 12

Table S4. Genetic instruments of Two-Sample Mendelian Randomization (Drinks per week, AUD and PAU) 14

Table S5. Results of the MR-PRESSO analysis. 19

Table S6. Genetic instruments associated with potential confounders in PhenoScanner. (European ancestry, P<5×10-8) 20

Table S7. Alcohol consumption-related CpG sites and mQTLs from ARIES database. 27

Table S8. Two-sample Mendelian Randomization estimates of methylation at alcohol consumption-related CpG sites on CRC risk. 30

Table S9. Characteristics of CRC incident cases and population-based controls nested in the UK Biobank. 32

Supplementary figures 33

Figure S1. Flowchart of the selection of eligible studies for meta-analysis. 33

CRC, colorectal cancer. 33

Figure S2. Pooled RR of CRC risk for light, moderate and heavy drinkers in the meta-analysis. Light, <12.5 g/d; Moderate, 12.5-50 g/d; Heavy, >50 g/d. 34

Figure S3. Dose-response effect of alcohol drinking on CRC risk. 35

Figure S4. Regional plots of cg10045354 and mapped genes. 35

Figure S5. Single-tissue eQTL plot of rs11213823 (cg10045354, COLCA2). 36

Figure S6. Single-tissue eQTL plot of rs11213823 (cg10045354, COLCA1). 37

Supplementary materials & methods

Detailed search strategy

MEDLINE

1 alcohols/
2 ethanol/
3 drinking/
4 drinking behavior/
5 exp alcohol drinking/
6 exp alcoholic beverages/
7 exp alcohol-related disorders/
8 ((alcohol and (consumption or drinking or intake)) or (alcohol abuse) or (alcoholic beverage*)).mp.
9 1 or 2 or 3 or 4 or 5 or 6 or 7 or 8
10 exp colorectal neoplasms/
11 exp colonic neoplasms/
12 exp rectal neoplasms/
13 ((colorectal or colonic or colon or rectal or rectum or bowel* or intestine* or sigmoid) and (cancer or neoplasm* or malign* or tumor* or tumour* or carcinom* or sarcom* or adenocarcinom* or adeno?carcinom* or adenom* or lesion*)).mp.
14 10 or 11 or 12 or 13
15 exp cohort studies/
16 cohort$.tw.
17 controlled clinical trial.pt.
18 15 or 16 or 17
19 9 and 14 and 18

Embase

1 alcohol/
2 alcohol consumption/
3 drinking/
4 drinking behavior/
5 alcoholism/
6 exp alcohol abuse/
7 exp alcoholic beverages/
8 ((alcohol and (consumption or drinking or intake)) or (alcohol abuse) or (alcoholic beverage*)).mp.
9 1 or 2 or 3 or 4 or 5 or 6 or 7 or 8
10 exp colon cancer/
11 exp rectum cancer/
12 ((colorectal or colonic or colon or rectal or rectum or bowel* or intestine* or sigmoid) and (cancer or neoplasm* or malign* or tumor* or tumour* or carcinom* or sarcom* or adenocarcinom* or adeno?carcinom* or adenom* or lesion*)).mp.

13 10 or 11 or 12
14 cohort analysis/
15 longitudinal study/
16 prospective study/
17 follow up/
18 cohort$.tw.
19 14 or 15 or 16 or 17 or 18
20 9 and 13 and 19

BIOSIS Citation index

1 TS=alcohols/
2 TS=ethanol/
3 TS=drinking/
4 TS=drinking behavior/
5 TS=exp alcohol drinking/
6 TS=exp alcoholic beverages/
7 TS=exp alcohol-related disorders/
8 TS= ((alcohol and (consumption or drinking or intake)) or (alcohol abuse) or (alcoholic beverage*)).mp.
9 1 or 2 or 3 or 4 or 5 or 6 or 7 or 8
10 TS=exp colorectal neoplasms/
11 TS=exp colonic neoplasms/
12 TS=exp rectal neoplasms/
13 TS= ((colorectal or colonic or colon or rectal or rectum or bowel* or intestine* or sigmoid) NEAR (cancer or neoplasm* or malign* or tumor* or tumour* or carcinom* or sarcom* or adenocarcinom* or adeno?carcinom* or adenom* or lesion*)).mp.
14 10 or 11 or 12 or 13
15 TS=cohort analysis/
16 TS=longitudinal study/
17 TS=prospective study/
18 TS=follow up/
19 TS=cohort$.tw.
20 15 or 16 or 17 or 18 or 19
21 9 and 14 and 20

Two-sample MR

Mendelian randomization (MR) design provides a cost-effective analogy to a randomized controlled trial by using genetic variants as proxies to test the causality of an association between exposure and outcome of interest. Here we applied inverse-variance weighted MR approach as the main analysis, and the simple mode, Egger, weighted median and weighted mode as sensitivity analyses to explore the robustness of the findings. Details of these MR approaches, including their different assumptions, are explained as

below.

Mean-based methods: The inverse variance weighted (IVW) MR and Egger MR provide two mean-based estimators. The IVW MR approach assumes that variants exhibit no horizontal pleiotropy, while Egger regression relaxes the horizontal pleiotropy assumption further by allowing a non-zero intercept which essentially allows overall horizontal pleiotropy to be directional, where its total effect influences the outcome in a specific direction. Egger regression further allows heterogeneity around the slope having accounted for overall directional horizontal pleiotropy, as long as the horizontal pleiotropy effects are not correlated with the SNP-exposure effects (also known as the INSIDE assumption).

Median-based methods: This analytical approach takes the median effect of all available instruments. The sample median method requires that half the instruments need to be valid to obtain unbiased estimate. The weighted median method allows stronger instruments to contribute more towards the estimate and obtain an estimate by weighting the contribution of each instrument by the inverse of its variance.

Mode-based methods: The mode-based estimator clusters the instruments into groups based on similarity of causal effects, and returns the final causal effect estimate based on the cluster that has the largest number of instruments. This provide two mode-based estimators: the simple mode is the unweighted mode of the empirical density function of causal estimates, the weighted mode is weighted by the inverse variance of the outcome effect.

MR-PRESSO: The MR-PRESSO is applied for the global test, outlier test, and distortion test using the MR pleiotropy residual sum and outlier (MR-PRESSO) R package. Specifically, the global test detects horizontal pleiotropy among the MR instruments; the outlier test corrects for horizontal pleiotropy via outlier removal; the distortion test identifies significant distortion in the causal estimates before and after outlier removal.

Supplementary tables

Table S1. Summarized characteristics of included studies.

Characteristics		No. of studies	Proportion (%)
Outcome	Incidence	25	78.1
	Mortality	7	21.9
Geographic area	North America	11	34.4
	Europe	9	28.1
	Asia	10	31.3
	Others/Mixed	2	6.3
Sex	M	5	15.6
	W	2	6.3
	M+W	15	46.8
	T	10	31.2
Anatomical site	C	3	9.4
	R	0	0.0
	C+R	11	34.4
	CR	18	56.3
Reference category	Without ex-drinker	10	31.3
	With ex-drinker/Unknown	22	68.8
	Without occasional drinker	24	75.0
	With occasional drinker	8	25.0

Table S2. Detailed characteristics of included studies.

Author and year	Population				Exposure assessment			Outcome evaluation			
	Cohort description and country	Sample size	Age	Sex	Ex-drinker in reference group	Occasional drinker in reference group	Adjusted factors	Follow-up	Outcome	Site	Events
Bamia 2013	European Prospective Investigation into Cancer and nutrition (EPIC), Mixed (Denmark, France, Germany, Greece, Italy, Netherlands, Norway, Spain, Sweden, and UK)	480308	Unknown	M, W, T	Unknown	N	sex, age at enrolment,body mass index, physical activity, educational level, smoking status at enrolment	11.6 years on average	Incidence	CR	4355
Betts 2018	Health and Lifestyle Survey (HALS1), UK	6 721	M: 47.90 (17.90); W: 48.00 (18.02)	M, W	N	N	Ethnicity, income, self-rated health, smoking status, BMI, and exercise	1984-2009	Incidence	CR	117
Bradbury 2020	UK Biobank, UK	473 138	40-69	T	Unknown	Y	Education, smoking status, waist circumference, height, physical activity, family history of CRC, aspirin or ibuprofen use, use of vitamin D supplements, use of folate supplements. For women only: parity, menopause, oral contraceptive agent and HRT use	2006-2014	Incidence	C, R, CR	2 599 (1 735 C, 864 R)
Breslow 2011	National Health Interview Survey (NHIS), USA	323 354	>=18	M, W, T	N	N	Race/ethnicity, education, region, marital status, smoking status, BMI, and gender	1988-2006	Mortality	CR	850

Buron Pust 2017	Million Women Study, UK	1 310 390	56.1 (4.6)	W	Unknown	Y	Socioeconomic status, height, BMI, smoking, strenuous exercise, age at menarche, births, hysterectomy, sterilisation, age at menopause, oral contraceptive use, and HRT use	1988-2013	Incidence	C, R, CR	18 518 (12 761 C, 5 757 R)
Cho 2004	Pooling Project of Prospective Studies of Diet and Cancer, Mixed (Canada, Netherlands, Sweden, and USA)	489 979	15-107	M, W, T	Unknown	N	Smoking, BMI, education, height, degree of physical activity, family history of colorectal cancer, use of nonsteroidal anti-inflammatory drugs, use of multivitamins, energy intake, red meat intake, total milk intake, folate intake from food only. For women only: history of use of oral contraceptives and postmenopausal HRT	1980-1998	Incidence	C, R, CR	4 687 (3 291 C, 1370 R)
Cho 2015	Korean Multi-center Cancer Cohort, Korea	18 522	>=20	M, W	N	N	BMI, moderate physical activity, and cigarette smoking history	1993-2011	Incidence	CR	220
Choi 2017	National Health Insurance Corporation (NHIC), Korea	23 179 312	>=20	M, W, T	Unknown	N	Age, sex, exercise, income, BMI, diabetes mellitus, and smoking status	2009-2012	Incidence	CR	154 868
Chyou 1996	Japanese-American men in Hawaii, USA	7 945	>=45	M	Unknown	N	Age	1965-1995	Incidence	C, R	453 (330 C, 123 R)
Flood 2002	Breast Cancer Detection Demonstration Project (BCDDP), USA	45 264	61.9 (40-93)	W	Unknown	N	Energy, dietary folate, methionine, and smoking	1987-1998	Incidence	CR	490

Gaziano 2000	Physicians' Health Study, USA	89 299	40-84	M	Y	Y	Age, smoking, diabetes, exercise and BMI	1982-1989	Mortality	C	101
Hippisley-Cox 2015	QResearch database, UK	4 943 765	25-84	M, W	Unknown	N	Age, BMI, Townsend score, ethnic group, smoking status, family history of colorectal cancer, ulcerative colitis, colonic polyp, and prior history of other cancer	1998-2013	Incidence	CR	32 626
Jayasekara 2017	Melbourne Collaborative Cohort Study (MCCS), Australia	38 149	40-69, 55.2 (8.6)	M, W, T	N	N	Sex, education, socioeconomic status, smoking, physical activity, energy intake from food, dietary fiber, dietary folate and total red meat	1990-2008	Incidence	C, R, CR	922 (596 C, 326 R)
Jo 2019	Korean Genome and Epidemiology Study (KoGES), Korea	2 327	40-69	T	N	N	Age, gender, living with spouse, educational level, income level, subjective health status, BMI, and cigarette smoking	2001-2014	Incidence	C	46
Klatsky 2015	Northern California Kaiser Permanente, USA	124 193	41	T	N	N	Age, sex, race/ethnicity, BMI, education, marital status, and smoking	1978-2012	Incidence	CR	2 148
Kono 1987	Male physicians in Western Japan, Japan	5 130	49 (27-89)	M	Y	Y	Age and smoking	1965-1983	Mortality	CR	39

Kunzmann 2018	Prostate, Lung, Colorectal, and Ovarian (PLCO) Cancer Screening Trial, USA	99 654	65 (10)	T	N	N	Study centre, race, BMI, randomisation group, smoking status by pack-years, year of DHQ completion, marital status, educational attainment, family history of cancer, HRT use, coffee intake, energy intake, red meat intakes, processed meat intakes, fruit and vegetable intake, dietary fibre intake and total calcium intake	1998-2009	Incidence	CR	944
Lim 2008	Korea Elderly Pharmacoepidemiologic Cohort (KEPEC), Korea	14 304	>=65	T	Unknown	N	Age and gender	1993-2002	Incidence	CR	112
Mizoue 2008	Pooled analysis of five cohort studies, Japan	209 763	>=35	M, W	Unknown	N	Age, smoking, BMI, intakes of energy, red meat, calcium, fiber, and folate, and area (adjusted in three cohorts)	1988-2004	Incidence	C, R, CR	2 802 (1 829 C, 967 R)
Park 2019	Multiethnic Cohort Study (MEC), USA	190 698	45-75	M, W, T	Unknown	N	Age at cohort entry, sex, race/ethnicity, family history of CRC, history of colorectal polyps, BMI, pack-years of cigarette smoking, multivitamin use, NSAID use, physical activity, menopausal HRT use, and daily intakes of total energy, red meat, dietary fiber, calcium, folate, and vitamin D	1993-2013	Incidence	C, R, CR	4 923 (3 257 C, 988 R)

Pedersen 2003	Copenhagen Centre for Prospective Population Studies, Denmark	426 934	Unknown	T	Unknown	Y	Age, sex, smoking, BMI, and study of origin	1976-1993	Incidence	C, R	613 (411 C, 202 R)
Sanjoaquin 2004	Oxford Vegetarian Study, UK	11 140	33 (median)	T	Unknown	Y	Age, sex, and smoking	1980-1999	Incidence	CR	95
Shen 2013	18 elderly health centres in Hong Kong, China	66 820	>=65	M, W, T	N	N	Age, education, housing type, monthly expenditure, BMI, exercise, health status, smoking status and smoking×age	1998-2012	Mortality	CR	944
Su 2004	Nutrition Examination Survey (NHANES I) Epidemiologic Follow-up Study (NHEFS), USA	10 418	M: 58.5 (14.7); W: 56.1 (14.8)	T	N	N	Age, sex, race, BMI, educational level, nonpoultry meat consumption, poultry and seafood consumption, regular multivitamin use, history of colonic polyps, and smoking status	1982-1993	Incidence	CR	111
Thun 1997	Cancer Prevention Study II, USA	457 423	56 (30-104)	M, W	N	N	Education, BMI, smoking, a crude index of fat consumption, and vegetable consumption	1982-1991	Mortality	CR	1 427
Toriola 2008	Findrink study, Finland	2 682	42-61	M	Unknown	Y	Age, examination year, vegetable consumption, fibre intake, family history of cancer, smoking, socio-economic status and leisure time physical activity	1984-2005	Incidence	CR	59

Tsong 2007	Singapore Chinese Health Study, Singapore	61 321	45-74	T	Unknown	N	Age, gender, dialect group, year of recruitment, level of education, BMI, history of diabetes, family history of colorectal cancer, cigarette smoking level, and physical exercise	1993-2004	Incidence	C, R, CR	845 (516 C, 329 R)
Tverdal 2021	Norwegian population-based health surveys, year 40 programme and the cohort of Norway, Norway	250 010	20-79	M, W	Unknown	N	Age, HDL-cholesterol, total cholesterol, triglycerides, number of cigarettes/day, systolic blood pressure, education, BMI, height, physical activity, and marriage status	18 years on average	Incidence	C, R	4 462 (3 023 C, 1 429 R)
Viner 2019	Alberta's Tomorrow Project (ATP), Canada	26 607	35-69	M, W	Unknown	N	Age, sex, BMI, marital status, highest level of education, total household income, smoking status, pack-years of cigarettes, and history of colon cancer screening	2001-2017	Incidence	C	192
Wu 1987	A retirement community south of Los Angeles, USA	11 644	Unknown	M, W	Unknown	Y	Age	1981-1985	Incidence	CR, C	126
Yang 2012	45 China's national Disease Surveillance Points, China	218 189	54.3 (40-79)	M	Unknown	N	Age, area, smoking and education	1990-2006	Mortality	CR	256
Yi 2020	Kangwha Cohort, Korea	6 291	>=55	T	Unknown	N	Age, history of chronic disease, smoking habit, ginseng intake, pesticide use, BMI, and education status	1985-2005	Mortality	C, R, CR	42 (29 C, 13 R)

Table S3. Quality assessment of included studies using the Newcastle-Ottawa Scale.

Author and year	Selection				Comparability		Outcome			Total quality score
	Representativeness of exposed cohort	Selection of non-exposed cohort	Ascertainment of exposure	Demonstration that outcome of interest was not present at start of study	Adjust for the most important risk factors [a]	Adjust for other risk factors [b]	Assessment of outcome	Follow-up length [c]	Loss to follow-up rate [d]	
Bamia 2013	1	1	1	1	1	1	1	1	1	9
Betts 2018	1	1	1	1	1	1	1	1	1	9
Bradbury 2020	1	1	1	1	1	1	1	1	1	9
Breslow 2011	1	1	1	1	1	1	1	1	1	9
Buron Pust 2017	1	1	1	1	1	1	1	1	1	9
Cho 2004	0	1	1	1	1	1	1	1	1	8
Cho 2015	1	1	1	1	1	1	1	1	1	9
Choi 2017	1	1	1	1	1	1	1	1	1	9
Chyou 1996	1	1	1	1	0	0	1	1	1	7
Flood 2002	0	1	1	1	0	0	1	1	1	6
Gaziano 2000	0	1	1	1	1	1	1	1	1	8
Hippisley-Cox 2015	1	1	1	1	1	1	1	0	1	8
Jayasekara 2017	1	1	1	1	0	1	1	1	1	8
Jo 2019	0	1	1	1	1	1	1	1	1	8
Klatsky 2015	1	1	1	1	1	1	1	1	1	9
Kono 1987	0	1	1	1	0	0	1	1	1	6
Kunzmann 2018	0	1	1	1	1	0	1	1	1	7
Lim 2008	1	1	1	1	0	0	1	0	1	6
Mizoue 2008	1	1	1	1	1	1	1	1	1	9
Park 2019	1	1	1	1	1	1	1	1	1	9

Pedersen 2003	1	1	1	1	1	1	1	1	1	9
Sanjoaquin 2004	0	1	1	1	0	1	1	1	1	7
Shen 2013	1	1	1	1	1	1	1	1	1	9
Su 2004	1	1	1	1	1	1	1	1	1	9
Thun 1997	1	1	1	1	1	1	1	1	1	9
Toriola 2008	1	1	1	1	0	1	1	1	1	8
Tsong 2007	1	1	1	1	1	1	1	1	1	9
Tverdal 2021	1	1	1	1	1	1	1	1	1	9
Viner 2019	1	1	1	1	1	1	1	1	1	9
Wu 1987	1	1	1	1	0	0	1	0	1	6
Yang 2012	1	1	1	1	0	1	1	1	1	8
Yi 2020	1	1	1	1	1	1	1	1	1	9

a Age, sex, and BMI.

b Other factors that are associated with colorectal cancer, including physical activity, smoking, red meat consumption, etc.

c Longer than five years follow-up.

d Follow-up rate more than 80%.

Table S4. Genetic instruments of Two-Sample Mendelian Randomization (Drinks per week, AUD and PAU).

Phenotype	SNP	Chr	Pos	beta	SE	P value	Effect allele	Other allele	EAF	F Statistic
Drinks Per Week	rs705687	1	4548453	-0.010904	0.001776	8.15E-10	G	A	0.785	37.7
Drinks Per Week	rs58107686	1	33837334	-0.009747	0.001585	7.79E-10	A	C	0.328	37.8
Drinks Per Week	rs12088813	1	66407700	-0.009329	0.001649	1.58E-08	C	A	0.267	32
Drinks Per Week	rs5024204	1	71491890	0.009703	0.001628	2.55E-09	T	A	0.278	35.5
Drinks Per Week	rs10753661	1	165119792	-0.008638	0.001569	3.76E-08	A	G	0.684	30.3
Drinks Per Week	rs28680958	1	173848808	-0.010996	0.00177	5.13E-10	A	G	0.217	38.6
Drinks Per Week	rs823114	1	205719532	0.008768	0.001467	2.31E-09	A	G	0.553	35.7
Drinks Per Week	rs77165542	2	430975	-0.026011	0.003971	5.63E-11	T	C	0.035	42.9
Drinks Per Week	rs1260326	2	27730940	0.02089	0.001488	8.05E-45	C	T	0.601	197
Drinks Per Week	rs13383034	2	45155276	0.014927	0.001551	6.31E-22	T	C	0.329	92.6
Drinks Per Week	rs13032049	2	63581507	0.010195	0.001618	3.00E-10	G	A	0.283	39.7
Drinks Per Week	rs828867	2	74334462	0.008757	0.001464	2.15E-09	A	G	0.545	35.8
Drinks Per Week	rs11692435	2	98275354	0.01745	0.002616	2.53E-11	A	G	0.085	44.5
Drinks Per Week	rs13024996	2	144225215	-0.010913	0.001515	5.72E-13	A	C	0.364	51.9
Drinks Per Week	rs72859280	2	147956293	0.022885	0.003902	4.44E-09	T	G	0.036	34.4
Drinks Per Week	rs56337305	2	225475560	-0.009588	0.001499	1.63E-10	C	T	0.383	40.9
Drinks Per Week	rs13094887	3	70968431	-0.01031	0.001589	8.57E-11	T	A	0.301	42.1
Drinks Per Week	rs62250685	3	85457240	-0.014357	0.0015	1.05E-21	G	A	0.614	91.6
Drinks Per Week	rs13066454	3	93994255	-0.008776	0.001492	4.13E-09	T	C	0.398	34.6
Drinks Per Week	rs9838144	3	131576287	-0.009964	0.001793	2.65E-08	C	G	0.209	30.9
Drinks Per Week	rs2011092	3	141124607	-0.0089	0.00154	7.35E-09	C	T	0.339	33.4
Drinks Per Week	rs6787172	3	158187811	-0.008031	0.001466	4.27E-08	G	T	0.554	30
Drinks Per Week	rs3748034	4	3446091	-0.01174	0.002082	1.67E-08	T	G	0.143	31.8
Drinks Per Week	rs11940694	4	39414993	0.02595	0.001486	3.03E-68	G	A	0.597	305
Drinks Per Week	rs4501255	4	42151306	0.010693	0.001719	4.83E-10	G	C	0.235	38.7
Drinks Per Week	rs1229984	4	100239319	0.150534	0.003861	<2.2E-308	C	T	0.963	1520
Drinks Per Week	rs36052336	4	100273594	-0.018429	0.003034	1.23E-09	G	A	0.061	36.9
Drinks Per Week	rs2165670	4	100286085	0.02308	0.002364	1.67E-22	A	G	0.106	95.3

Drinks Per Week	rs79139602	4	100444363	0.060272	0.005076	1.80E-32	T	A	0.021	141
Drinks Per Week	rs4699791	4	101243023	0.018571	0.002477	6.58E-14	A	G	0.096	56.2
Drinks Per Week	rs13107325	4	103188709	-0.027505	0.002816	1.53E-22	T	C	0.072	95.4
Drinks Per Week	rs4690727	4	143648579	0.010817	0.00162	2.43E-11	G	C	0.718	44.6
Drinks Per Week	rs12651313	4	171086393	-0.008643	0.001467	3.79E-09	G	C	0.443	34.7
Drinks Per Week	rs4916723	5	87854395	-0.009952	0.001479	1.72E-11	C	A	0.416	45.3
Drinks Per Week	rs12655091	5	144412335	-0.008312	0.00146	1.25E-08	A	G	0.53	32.4
Drinks Per Week	rs55872084	5	155902003	0.009979	0.001719	6.32E-09	T	G	0.235	33.7
Drinks Per Week	rs6460047	7	73042443	0.011624	0.001796	9.69E-11	C	T	0.208	41.9
Drinks Per Week	rs10236149	7	98977515	-0.013498	0.002219	1.18E-09	G	A	0.123	37
Drinks Per Week	rs35034355	7	103840115	-0.008097	0.001459	2.87E-08	A	G	0.521	30.8
Drinks Per Week	rs6951574	7	153489744	0.013222	0.001463	1.58E-19	C	T	0.458	81.7
Drinks Per Week	rs13250583	8	20949917	-0.009718	0.00178	4.70E-08	T	C	0.213	29.8
Drinks Per Week	rs1217091	8	64527399	0.012161	0.001865	7.05E-11	C	T	0.812	42.5
Drinks Per Week	rs28601761	8	126500031	0.009103	0.001477	7.17E-10	G	C	0.42	38
Drinks Per Week	rs55932213	9	108755622	0.009488	0.001654	9.55E-09	G	A	0.736	32.9
Drinks Per Week	rs10978550	9	109345993	-0.011748	0.001802	7.15E-11	C	T	0.206	42.5
Drinks Per Week	rs7074871	10	110507806	-0.0094	0.001672	1.86E-08	A	G	0.255	31.6
Drinks Per Week	rs17665139	10	125093880	-0.01156	0.002047	1.59E-08	T	C	0.149	31.9
Drinks Per Week	rs7950166	11	8642218	-0.009799	0.001516	9.89E-11	T	C	0.637	41.8
Drinks Per Week	rs11030084	11	27643725	-0.010607	0.001881	1.72E-08	T	C	0.184	31.8
Drinks Per Week	rs56030824	11	47397353	-0.011602	0.001563	1.15E-13	A	G	0.322	55.1
Drinks Per Week	rs10750025	11	113424042	0.010321	0.00157	4.89E-11	T	C	0.686	43.2
Drinks Per Week	rs1713676	11	113660576	-0.007992	0.001459	4.29E-08	G	A	0.522	30
Drinks Per Week	rs4938230	11	116075001	0.01281	0.001998	1.48E-10	A	C	0.842	41.1
Drinks Per Week	rs682011	11	121544285	0.008212	0.001468	2.22E-08	C	T	0.559	31.3
Drinks Per Week	rs12795042	11	133658168	-0.008319	0.001504	3.25E-08	C	A	0.623	30.6
Drinks Per Week	rs10876188	12	51895882	-0.007987	0.001463	4.84E-08	T	C	0.457	29.8
Drinks Per Week	rs3809162	12	54674235	0.009061	0.00149	1.19E-09	G	A	0.397	37
Drinks Per Week	rs10506274	12	81601464	-0.009037	0.001458	5.78E-10	T	G	0.484	38.4
Drinks Per Week	rs4842786	12	92170791	-0.008798	0.001479	2.73E-09	A	G	0.584	35.4
Drinks Per Week	rs500321	13	27124360	-0.009669	0.001653	4.92E-09	T	A	0.736	34.2

Drinks Per Week	rs1123285	14	57274519	-0.008897	0.001544	8.14E-09	G	C	0.335	33.2
Drinks Per Week	rs2180870	14	58782779	-0.012178	0.002133	1.12E-08	C	T	0.135	32.6
Drinks Per Week	rs28929474	14	94844947	-0.0368	0.005438	1.34E-11	T	C	0.018	45.8
Drinks Per Week	rs11625650	14	104610138	-0.009568	0.001724	2.89E-08	A	G	0.233	30.8
Drinks Per Week	rs2472297	15	75027880	0.010606	0.001685	3.10E-10	T	C	0.249	39.6
Drinks Per Week	rs12907323	15	86796012	0.008497	0.001481	9.93E-09	G	A	0.411	32.9
Drinks Per Week	rs2764771	16	20013793	0.009891	0.001582	4.02E-10	A	G	0.307	39.1
Drinks Per Week	rs17177078	16	24810681	-0.022315	0.003012	1.27E-13	T	C	0.063	54.9
Drinks Per Week	rs378421	16	28754684	-0.011205	0.001487	4.83E-14	A	G	0.404	56.8
Drinks Per Week	rs113443718	16	29892184	-0.010208	0.001585	1.19E-10	A	G	0.305	41.5
Drinks Per Week	rs62044525	16	64872590	-0.012173	0.001883	1.03E-10	G	C	0.184	41.8
Drinks Per Week	rs7185555	16	69131281	-0.011101	0.002027	4.24E-08	C	G	0.153	30
Drinks Per Week	rs79616692	16	72338507	0.016302	0.002351	4.11E-12	C	G	0.108	48.1
Drinks Per Week	rs1104608	16	73912588	-0.010975	0.001476	1.05E-13	C	G	0.425	55.3
Drinks Per Week	rs4548913	17	2209888	-0.00836	0.001511	3.11E-08	A	G	0.632	30.6
Drinks Per Week	rs3803800	17	7462969	0.011379	0.001777	1.50E-10	G	A	0.786	41
Drinks Per Week	rs2854334	17	29715500	0.009221	0.001498	7.51E-10	G	A	0.615	37.9
Drinks Per Week	rs2532276	17	44246624	-0.0218	0.002559	1.62E-17	A	C	0.215	72.6
Drinks Per Week	rs10438820	17	78524597	0.008972	0.001593	1.76E-08	T	C	0.702	31.7
Drinks Per Week	rs9950000	18	53052169	-0.009118	0.001491	9.38E-10	T	C	0.395	37.4
Drinks Per Week	rs4092465	18	55080437	-0.008292	0.001514	4.39E-08	G	A	0.635	30
Drinks Per Week	rs281379	19	49214274	0.013722	0.001458	4.91E-21	A	G	0.508	88.6
Drinks Per Week	rs4815364	20	25035711	0.008582	0.001499	1.02E-08	A	G	0.616	32.8
Drinks Per Week	rs9607814	22	41946519	-0.010181	0.001859	4.31E-08	A	C	0.2	30
Alcohol Use Disorder	rs12743648	1	66428502	-0.023427	0.004014	5.34E-09	T	G	0.234	61.82
Alcohol Use Disorder	rs494904	2	45141180	-0.024796	0.003535	2.32E-12	T	C	0.589	93.46
Alcohol Use Disorder	rs1402398	2	58042241	0.020785	0.003499	2.85E-09	A	G	0.628	63.39
Alcohol Use Disorder	rs13411140	2	144215811	-0.019358	0.003495	3.03E-08	T	C	0.363	54.4
Alcohol Use Disorder	rs2713557	2	227171603	0.019781	0.00352	1.92E-08	T	C	0.356	56.35
Alcohol Use Disorder	rs13129401	4	39404872	-0.023406	0.003416	7.33E-12	A	G	0.454	85.28
Alcohol Use Disorder	rs1229984	4	100239319	-0.223527	0.01016	2.88E-107	T	C	0.03	921.56
Alcohol Use Disorder	rs13107325	4	103188709	-0.056092	0.006328	7.71E-19	T	C	0.079	144.14

Alcohol Use Disorder	rs12192123	6	31459342	-0.02216	0.003962	2.23E-08	A	G	0.232	54.97
Alcohol Use Disorder	rs10270358	7	114948351	0.021961	0.003636	1.55E-09	A	G	0.318	65.71
Alcohol Use Disorder	rs4240624	8	9184231	0.026018	0.00447	5.88E-09	A	G	0.913	33.87
Alcohol Use Disorder	rs7073987	10	110565868	-0.022452	0.003877	7.01E-09	T	C	0.259	60.72
Alcohol Use Disorder	rs7117878	11	57421457	0.021106	0.003662	8.26E-09	A	C	0.316	60.5
Alcohol Use Disorder	rs6589386	11	113443753	-0.023179	0.003381	7.08E-12	T	C	0.424	82.4
Alcohol Use Disorder	rs61974485	14	58765903	0.021868	0.003974	3.74E-08	T	C	0.266	58.67
Alcohol Use Disorder	rs8008020	14	104355883	0.019256	0.003419	1.78E-08	T	C	0.416	56.58
Alcohol Use Disorder	rs73403005	15	47645174	-0.020087	0.003454	6.06E-09	A	G	0.841	33.81
Alcohol Use Disorder	rs9926289	16	53820503	-0.024484	0.003413	7.28E-13	A	G	0.398	90.19
Problematic Alcohol Use	rs6421482	1	66419905	-0.016437	0.002603	2.70E-10	A	G	0.436	57.89
Problematic Alcohol Use	rs1260326	2	27730940	-0.024563	0.002642	1.45E-20	T	C	0.403	126.52
Problematic Alcohol Use	rs494904	2	45141180	-0.021371	0.002696	2.26E-15	T	C	0.596	95.81
Problematic Alcohol Use	rs1402398	2	58042241	0.019088	0.002689	1.27E-12	A	G	0.627	74.21
Problematic Alcohol Use	rs9679319	2	104134432	-0.015647	0.002604	1.86E-09	T	G	0.48	53.24
Problematic Alcohol Use	rs13382553	2	138264231	-0.018415	0.003069	1.97E-09	A	G	0.766	52.95
Problematic Alcohol Use	rs2673136	2	227164653	-0.015802	0.002691	4.31E-09	A	G	0.639	50.2
Problematic Alcohol Use	rs62250713	3	85513793	0.016267	0.002689	1.46E-09	A	G	0.368	53.62
Problematic Alcohol Use	rs13129401	4	39404872	-0.023316	0.002618	5.29E-19	A	G	0.453	117.39
Problematic Alcohol Use	rs2602856	4	100029145	-0.015678	0.002732	9.56E-09	A	C	0.661	47.99
Problematic Alcohol Use	rs1229984	4	100239319	-0.223527	0.01016	2.88E-107	T	C	0.03	1278.5
Problematic Alcohol Use	rs13135092	4	103198082	0.056302	0.004823	1.75E-31	A	G	0.919	205.19
Problematic Alcohol Use	rs2582405	8	57424874	0.017499	0.003043	8.86E-09	T	C	0.237	48.24
Problematic Alcohol Use	rs7900002	10	72907951	-0.014549	0.002644	3.74E-08	T	G	0.601	44.22
Problematic Alcohol Use	rs56722963	10	110537834	-0.019028	0.002985	1.85E-10	T	C	0.255	59.94
Problematic Alcohol Use	rs576859	11	57480623	0.020327	0.003585	1.43E-08	A	C	0.327	79.25
Problematic Alcohol Use	rs6589386	11	113443753	-0.019549	0.002603	5.88E-14	T	C	0.432	81.72
Problematic Alcohol Use	rs10790456	11	121624837	0.023175	0.004123	1.89E-08	A	G	0.78	80.3
Problematic Alcohol Use	rs12296477	12	51903860	0.014307	0.002609	4.15E-08	C	G	0.547	44.19
Problematic Alcohol Use	rs61974485	14	58765903	0.016631	0.00302	3.67E-08	T	C	0.265	46.89
Problematic Alcohol Use	rs8008020	14	104355883	0.015959	0.002633	1.35E-09	T	C	0.418	53.96
Problematic Alcohol Use	rs72768626	16	24693048	0.03219	0.005757	2.26E-08	A	G	0.945	47.08

Problematic Alcohol Use	rs9937709	16	53820813	0.017382	0.002633	4.06E-11	A	G	0.585	63.9
Problematic Alcohol Use	rs492602	19	49206417	-0.015998	0.002604	8.08E-10	A	G	0.508	55.73

Table S5. Results of the MR-PRESSO analysis.

Phenotype	No. of SNP	Outlier	Global test. P value	Distortion test. P value	MR analysis	Beta	SE	P Value
Drinks per week	84	1	5.00E-04	0.716	Raw	0.581	0.192	0.003
					Outlier-corrected	0.656	0.177	3.90E-04
AUD	18	0	0.141	0.079	Raw	0.284	0.169	0.111
					Outlier-corrected	-	-	-
PAU	24	1	0.002	0.568	Raw	0.424	0.206	0.052
					Outlier-corrected	0.336	0.177	0.071

Table S6. Genetic instruments associated with potential confounders in PhenoScanner. (European ancestry, P<5×10-8)

Phenotype	SNP	Chr	Pos	A1	A2	Trait	Study	Beta	SE	P	N	Unit
Drinks per week	rs58107686	1	33837334	A	C	Hip circumference	Neale B	-0.01647	0.00256	1.24E-10	336601	IVNT
Drinks per week	rs58107686	1	33837334	A	C	Weight	Neale B	-0.01794	0.002259	1.95E-15	336227	IVNT
Drinks per week	rs823114	1	205719532	A	G	Weight	Neale B	-0.01249	0.002129	4.47E-09	336227	IVNT
Drinks per week	rs77165542	2	430975	C	T	Body mass index	Neale B	0.09532	0.00657	1.12E-47	336107	IVNT
Drinks per week	rs77165542	2	430975	C	T	Hip circumference	Neale B	0.08644	0.006579	2.03E-39	336601	IVNT
Drinks per week	rs77165542	2	430975	C	T	Waist circumference	Neale B	0.07051	0.005884	4.45E-33	336639	IVNT
Drinks per week	rs77165542	2	430975	C	T	Weight	Neale B	0.08737	0.005804	3.37E-51	336227	IVNT
Drinks per week	rs1260326	2	27730940	C	T	Inflammatory bowel disease	IBDGC	-0.07711	0.009964	1.00E-14	-	log OR
Drinks per week	rs1260326	2	27730940	C	T	Weight	Neale B	0.01778	0.00217	2.53E-16	336227	IVNT
Drinks per week	rs13383034	2	45155276	C	T	Ever smoked	Neale B	-0.007121	0.001293	3.67E-08	336067	risk diff
Drinks per week	rs13024996	2	144225215	A	C	Years of educational attainment	SSGAC	0.018	0.003	1.85E-12	328917	years
Drinks per week	rs62250685	3	85457240	A	G	Ever smoked	Neale B	0.0091	0.001225	1.10E-13	336067	risk diff
Drinks per week	rs62250685	3	85457240	A	G	Past tobacco smoking	Neale B	-0.02674	0.003263	2.48E-16	310749	-
Drinks per week	rs62250685	3	85457240	A	G	Smoking status: previous	Neale B	0.008552	0.001198	9.57E-13	336024	risk diff
Drinks per week	rs62250685	3	85457240	A	G	Body mass index	Neale B	0.01651	0.002476	2.60E-11	336107	IVNT
Drinks per week	rs62250685	3	85457240	A	G	Types of physical activity in last 4 weeks: other exercises	Neale B	0.00704	0.001257	2.16E-08	335599	risk diff
Drinks per week	rs62250685	3	85457240	A	G	Weight	Neale B	0.01583	0.002187	4.51E-13	336227	IVNT
Drinks per week	rs13066454	3	93994255	C	T	Body mass index	Neale B	0.01995	0.002455	4.36E-16	336107	IVNT
Drinks per week	rs13066454	3	93994255	C	T	Hip circumference	Neale B	0.01392	0.002458	1.49E-08	336601	IVNT
Drinks per week	rs13066454	3	93994255	C	T	Waist circumference	Neale B	0.01559	0.002198	1.32E-12	336639	IVNT
Drinks per week	rs13066454	3	93994255	C	T	Weight	Neale B	0.01474	0.002168	1.06E-11	336227	IVNT
Drinks per week	rs9838144	3	131576287	C	G	Body mass index	Neale B	0.0189	0.002974	2.08E-10	336107	IVNT
Drinks per week	rs9838144	3	131576287	C	G	Waist circumference	Neale B	0.01519	0.002662	1.15E-08	336639	IVNT
Drinks per week	rs9838144	3	131576287	C	G	Weight	Neale B	0.01647	0.002627	3.58E-10	336227	IVNT
Drinks per week	rs2011092	3	141124607	T	C	Hip circumference	GIANT	-0.031	0.0046	2.50E-11	145362	IVNT
Drinks per week	rs2011092	3	141124607	T	C	Weight	GIANT	-0.03311	0.00466	1.22E-12	125914	IVNT
Drinks per week	rs2011092	3	141124607	T	C	Hip circumference	Neale B	-0.02503	0.002529	4.40E-23	336601	IVNT
Drinks per week	rs2011092	3	141124607	T	C	Waist circumference	Neale B	-0.0141	0.002262	4.57E-10	336639	IVNT

Drinks per week	rs2011092	3	141124607	T	C	Weight	Neale B	-0.03049	0.002231	1.65E-42	336227	IVNT
Drinks per week	rs13107325	4	103188709	T	C	Body mass index	GIANT	0.058	0.009899	4.66E-09	126058	IVNT
Drinks per week	rs13107325	4	103188709	T	C	Body mass index	GIANT	0.0477	0.0068	1.83E-12	321461	IVNT
Drinks per week	rs13107325	4	103188709	T	C	Body mass index in physically active indivdiuals	GIANT	0.0531	0.0088	1.46E-09	132755	IVNT
Drinks per week	rs13107325	4	103188709	T	C	Body mass index in males greater than 50 years of age	GIANT	0.065	0.011	2.60E-09	90992	IVNT
Drinks per week	rs13107325	4	103188709	T	C	Body mass index adjusted for physical activity	GIANT	0.0505	0.0079	2.00E-10	173078	IVNT
Drinks per week	rs13107325	4	103188709	T	C	Body mass index adjusted for smoking	GIANT	0.0497	0.0077	8.98E-11	181290	IVNT
Drinks per week	rs13107325	4	103188709	T	C	Body mass index	GIANT	0.055	0.0044	2.80E-35	449889	IVNT
Drinks per week	rs13107325	4	103188709	T	C	Body mass index	Speliotes EK	NA	NA	1.50E-13	123865	-
Drinks per week	rs13107325	4	103188709	T	C	Body mass index	Speliotes EK	0.19	0.02585	2.00E-13	-	kg/m2 increase
Drinks per week	rs13107325	4	103188709	T	C	Body mass index	GIANT	0.0741	0.01191	5.00E-10	-	kg/m
Drinks per week	rs13107325	4	103188709	T	C	Crohns disease	IBDGC	0.2216	0.0397	2.40E-08	20883	log OR
Drinks per week	rs13107325	4	103188709	T	C	BMI adjusted for smoking behaviour	GIANT	0.0469	0.007446	3.00E-10	-	kg/m
Drinks per week	rs13107325	4	103188709	T	C	BMI adjusted for smoking behaviour	GIANT	0.0607	0.01032	4.00E-09	-	kg/m
Drinks per week	rs13107325	4	103188709	T	C	Body mass index	GIANT	0.0488	0.007748	3.00E-10	-	kg/m
Drinks per week	rs13107325	4	103188709	T	C	Body mass index	GIANT	0.0698	0.01142	1.00E-09	-	kg/m
Drinks per week	rs13107325	4	103188709	T	C	Body mass index	GIANT	0.0505	0.007939	2.00E-10	-	kg/m
Drinks per week	rs13107325	4	103188709	T	C	Body mass index	Neale B	0.0523	0.004572	2.66E-30	336107	IVNT
Drinks per week	rs13107325	4	103188709	T	C	Hip circumference	Neale B	0.04256	0.004578	1.45E-20	336601	IVNT
Drinks per week	rs13107325	4	103188709	T	C	Body mass index in physically active individuals	GIANT	0.0508	0.008633	4.00E-09	-	kg/m
Drinks per week	rs13107325	4	103188709	T	C	Body mass index in physically active individuals	GIANT	0.0531	0.008692	1.00E-09	-	kg/m
Drinks per week	rs13107325	4	103188709	T	C	Body mass index joint analysis main effects and physical activity interaction	GIANT	NA	NA	2.00E-09	-	-
Drinks per week	rs13107325	4	103188709	T	C	Body mass index joint analysis main effects and physical activity interaction	GIANT	NA	NA	2.00E-10	-	-
Drinks per week	rs13107325	4	103188709	T	C	Body mass index joint analysis main effects and physical activity interaction	GIANT	NA	NA	2.00E-08	-	-
Drinks per week	rs13107325	4	103188709	T	C	Body mass index joint analysis main effects and physical activity interaction	GIANT	NA	NA	8.00E-10	-	-
Drinks per week	rs13107325	4	103188709	T	C	Body mass index joint analysis main effects and smoking interaction	GIANT	NA	NA	2.00E-10	-	-

Drinks per week	rs13107325	4	103188709	T	C	Body mass index joint analysis main effects and smoking interaction	GIANT	NA	NA	1.00E-08	-	-
Drinks per week	rs13107325	4	103188709	T	C	Types of physical activity in last 4 weeks: strenuous sports	Neale B	-0.00829	0.001406	3.73E-09	335599	risk diff
Drinks per week	rs13107325	4	103188709	T	C	Waist circumference	Neale B	0.02421	0.004094	3.36E-09	336639	IVNT
Drinks per week	rs13107325	4	103188709	T	C	Weight	Neale B	0.02814	0.004039	3.24E-12	336227	IVNT
Drinks per week	rs13107325	4	103188709	T	C	Body mass index	Speliotes EK	NA	NA	2.00E-13	-	-
Drinks per week	rs28601761	8	126500031	C	G	Treatment with aspirin	Neale B	0.004867	0.0008424	7.56E-09	337159	risk diff
Drinks per week	rs28601761	8	126500031	C	G	Hip circumference	Neale B	-0.01941	0.002465	3.39E-15	336601	IVNT
Drinks per week	rs28601761	8	126500031	C	G	Weight	Neale B	-0.01796	0.002175	1.49E-16	336227	IVNT
Drinks per week	rs7950166	11	8642218	C	T	Body mass index	Neale B	-0.01715	0.00251	8.25E-12	336107	IVNT
Drinks per week	rs7950166	11	8642218	C	T	Waist circumference	Neale B	-0.01377	0.002247	8.97E-10	336639	IVNT
Drinks per week	rs7950166	11	8642218	C	T	Weight	Neale B	-0.01443	0.002217	7.70E-11	336227	IVNT
Drinks per week	rs11030084	11	27643725	T	C	Body mass index in females greater than 50 years of age	GIANT	-0.042	0.0067	2.80E-10	88424	IVNT
Drinks per week	rs11030084	11	27643725	T	C	Body mass index	Speliotes EK	NA	NA	7.37E-12	123865	IVNT
Drinks per week	rs11030084	11	27643725	T	C	Body mass index	GIANT	-0.04248	0.005689	8.21E-14	126576	IVNT
Drinks per week	rs11030084	11	27643725	T	C	Body mass index	GIANT	-0.0419	0.0048	2.57E-18	233665	IVNT
Drinks per week	rs11030084	11	27643725	T	C	Hip circumference	GIANT	-0.032	0.0057	2.00E-08	145105	IVNT
Drinks per week	rs11030084	11	27643725	T	C	Obesity class 1	GIANT	-0.099	0.016	1.00E-09	96739	log OR
Drinks per week	rs11030084	11	27643725	T	C	Overweight	GIANT	-0.078	0.012	1.80E-11	157563	log OR
Drinks per week	rs11030084	11	27643725	T	C	Waist circumference	GIANT	-0.034	0.0055	1.00E-09	153605	IVNT
Drinks per week	rs11030084	11	27643725	T	C	Weight	GIANT	-0.03556	0.00576	6.71E-10	125943	IVNT
Drinks per week	rs11030084	11	27643725	T	C	Body mass index	Speliotes EK	NA	NA	7.37E-12	123865	-
Drinks per week	rs11030084	11	27643725	T	C	Body mass index	Neale B	-0.0384	0.003079	1.05E-35	336107	IVNT
Drinks per week	rs11030084	11	27643725	T	C	Hip circumference	Neale B	-0.03276	0.003082	2.20E-26	336601	IVNT
Drinks per week	rs11030084	11	27643725	T	C	Waist circumference	Neale B	-0.02778	0.002757	6.93E-24	336639	IVNT
Drinks per week	rs11030084	11	27643725	T	C	Weight	Neale B	-0.02984	0.002719	5.11E-28	336227	IVNT
Drinks per week	rs11030084	11	27643725	T	C	Body mass index in males greater than 50 years of age	GIANT	-0.049	0.007	5.00E-12	73864	IVNT
Drinks per week	rs11030084	11	27643725	T	C	Body mass index in non-smokers	GIANT	-0.0383	0.0056	1.15E-11	129008	IVNT
Drinks per week	rs11030084	11	27643725	T	C	Body mass index adjusted for physical activity	GIANT	-0.0372	0.0054	5.77E-12	141741	IVNT
Drinks per week	rs11030084	11	27643725	T	C	Body mass index adjusted for smoking	GIANT	-0.038	0.0049	1.57E-14	163829	IVNT

Drinks per week	rs11030084	11	27643725	T	C	Past tobacco smoking	Neale B	0.02439	0.004056	1.83E-09	310749	-
Drinks per week	rs56030824	11	47397353	A	G	Body mass index	Neale B	0.02307	0.002573	3.10E-19	336107	IVNT
Drinks per week	rs56030824	11	47397353	A	G	Hip circumference	Neale B	0.01721	0.002576	2.37E-11	336601	IVNT
Drinks per week	rs56030824	11	47397353	A	G	Waist circumference	Neale B	0.01575	0.002303	7.98E-12	336639	IVNT
Drinks per week	rs2764771	16	20013793	A	G	Weight	Neale B	0.01442	0.00232	5.12E-10	336227	IVNT
Drinks per week	rs378421	16	28754684	G	A	Body mass index	Neale B	-0.02632	0.002451	6.75E-27	336107	IVNT
Drinks per week	rs378421	16	28754684	G	A	Hip circumference	Neale B	-0.03136	0.002453	2.08E-37	336601	IVNT
Drinks per week	rs378421	16	28754684	G	A	Types of physical activity in last 4 weeks: light diy	Neale B	0.009088	0.00124	2.32E-13	335599	risk diff
Drinks per week	rs378421	16	28754684	G	A	Waist circumference	Neale B	-0.02549	0.002194	3.41E-31	336639	IVNT
Drinks per week	rs378421	16	28754684	G	A	Weight	Neale B	-0.02659	0.002165	1.17E-34	336227	IVNT
Drinks per week	rs378421	16	28754684	G	A	Crohns disease	IBDGC	-0.1489	0.0254	4.67E-09	20883	log OR
Drinks per week	rs113443718	16	29892184	A	G	Weight	Neale B	0.01567	0.002299	9.19E-12	336227	IVNT
Drinks per week	rs4548913	17	2209888	A	G	Body mass index	Neale B	-0.01375	0.002511	4.34E-08	336107	IVNT
Drinks per week	rs2854334	17	29715500	A	G	Hip circumference	Neale B	0.01641	0.002483	3.89E-11	336601	IVNT
Drinks per week	rs2854334	17	29715500	A	G	Weight	Neale B	0.01321	0.002191	1.66E-09	336227	IVNT
Drinks per week	rs281379	19	49214274	A	G	Crohns disease	Franke A	NA	NA	7.40E-12	21389	-
Drinks per week	rs281379	19	49214274	A	G	Crohns disease	Anderson CA	NA	NA	8.60E-10	26405	-
Drinks per week	rs281379	19	49214274	A	G	Psoriasis and Crohns disease combined	Ellinghaus D	NA	NA	1.32E-17	15131	-
Drinks per week	rs281379	19	49214274	A	G	Crohns disease	IBDGC	NA	NA	8.60E-10	14342	log OR
Drinks per week	rs281379	19	49214274	A	G	Crohns disease	IBDGC	0.1398	0.0238	4.26E-09	20883	log OR
Drinks per week	rs281379	19	49214274	A	G	Crohns disease	Franke A	0.06766	0.009866	7.00E-12	-	log OR
Alcohol use disorder	rs494904	2	44914041	C	T	Ever smoked	Neale B	0.006818	0.001221	2.368E-08	336067	risk diff
Alcohol use disorder	rs494904	2	44914041	C	T	Past tobacco smoking	Neale B	-0.0187	0.003254	9.124E-09	310749	-
Alcohol use disorder	rs13411140	2	143458242	C	T	Qualifications: A levels or as levels or equivalent	Neale B	-0.007149	0.001129	2.422E-10	334070	risk diff
Alcohol use disorder	rs13411140	2	143458242	C	T	Qualifications: college or university degree	Neale B	-0.008655	0.001178	2.018E-13	334070	risk diff
Alcohol use disorder	rs13411140	2	143458242	C	T	Qualifications: none	Neale B	0.006449	0.000955	1.445E-11	334070	risk diff
Alcohol use disorder	rs13411140	2	143458242	C	T	Years of educational attainment	SSGAC	-0.018	0.003	1.541E-12	328917	years
Alcohol Use Disorder	rs13107325	4	102267552	T	C	Body mass index in physically active indivdiuals	GIANT	0.0531	0.0088	1.46E-09	132755	IVNT
Alcohol Use Disorder	rs13107325	4	102267552	T	C	Body mass index in males greater than 50 years of age	GIANT	0.065	0.011	2.6E-09	90992	IVNT
Alcohol Use Disorder	rs13107325	4	102267552	T	C	Body mass index adjusted for physical activity	GIANT	0.0505	0.0079	2E-10	173078	IVNT
Alcohol use disorder	rs13107325	4	102267552	T	C	Body mass index adjusted for smoking	GIANT	0.0497	0.0077	8.982E-11	181290	IVNT
Alcohol use disorder	rs13107325	4	102267552	T	C	Body mass index	GIANT	0.058	0.009899	4.66E-09	126058	IVNT

Alcohol use disorder	rs13107325	4	102267552	T	C	Body mass index	GIANT	0.0477	0.0068	1.825E-12	321461	IVNT
Alcohol use disorder	rs13107325	4	102267552	T	C	Body mass index	GIANT	0.055	0.0044	2.8E-35	449889	IVNT
Alcohol use disorder	rs13107325	4	102267552	T	C	Body mass index	Speliotes EK	NA	NA	1.5E-13	123865	-
Alcohol use disorder	rs13107325	4	102267552	T	C	Crohns disease	IBDGC	0.2216	0.0397	2.4E-08	20883	log OR
Alcohol use disorder	rs13107325	4	102267552	T	C	BMI adjusted for smoking behaviour	GIANT	0.0469	0.007446	3E-10	-	kg/m
Alcohol use disorder	rs13107325	4	102267552	T	C	BMI adjusted for smoking behaviour	GIANT	0.0607	0.01032	4E-09	-	kg/m
Alcohol use disorder	rs13107325	4	102267552	T	C	Body mass index	Speliotes EK	0.19	0.02585	2E-13	-	kg/m2 increase
Alcohol use disorder	rs13107325	4	102267552	T	C	Body mass index	GIANT	0.0741	0.01191	5E-10	-	kg/m
Alcohol use disorder	rs13107325	4	102267552	T	C	Body mass index	GIANT	0.0488	0.007748	3E-10	-	kg/m
Alcohol use disorder	rs13107325	4	102267552	T	C	Body mass index	GIANT	0.0698	0.01142	1E-09	-	kg/m
Alcohol use disorder	rs13107325	4	102267552	T	C	Body mass index	GIANT	0.0505	0.007939	2E-10	-	kg/m
Alcohol use disorder	rs13107325	4	102267552	T	C	Body mass index in physically active individuals	GIANT	0.0508	0.008633	4E-09	-	kg/m
Alcohol use disorder	rs13107325	4	102267552	T	C	Body mass index in physically active individuals	GIANT	0.0531	0.008692	1E-09	-	kg/m
Alcohol use disorder	rs13107325	4	102267552	T	C	Body mass index joint analysis main effects and physical activity interaction	GIANT	NA	NA	2E-09	-	-
Alcohol use disorder	rs13107325	4	102267552	T	C	Body mass index joint analysis main effects and physical activity interaction	GIANT	NA	NA	2E-10	-	-
Alcohol use disorder	rs13107325	4	102267552	T	C	Body mass index joint analysis main effects and physical activity interaction	GIANT	NA	NA	0.00000002	-	-
Alcohol use disorder	rs13107325	4	102267552	T	C	Body mass index joint analysis main effects and physical activity interaction	GIANT	NA	NA	8E-10	-	-
Alcohol use disorder	rs13107325	4	102267552	T	C	Body mass index joint analysis main effects and smoking interaction	GIANT	NA	NA	2E-10	-	-
Alcohol use disorder	rs13107325	4	102267552	T	C	Body mass index joint analysis main effects and smoking interaction	GIANT	NA	NA	0.00000001	-	-
Alcohol use disorder	rs13107325	4	102267552	T	C	Body mass index	Neale B	0.0523	0.004572	2.662E-30	336107	IVNT
Alcohol use disorder	rs13107325	4	102267552	T	C	Hip circumference	Neale B	0.04256	0.004578	1.449E-20	336601	IVNT
Alcohol use disorder	rs13107325	4	102267552	T	C	Qualifications: college or university degree	Neale B	-0.01236	0.002169	1.194E-08	334070	risk diff
Alcohol use disorder	rs13107325	4	102267552	T	C	Types of physical activity in last 4 weeks: strenuous sports	Neale B	-0.00829	0.001406	3.727E-09	335599	risk diff
Alcohol use disorder	rs13107325	4	102267552	T	C	Waist circumference	Neale B	0.02421	0.004094	3.355E-09	336639	IVNT
Alcohol use disorder	rs13107325	4	102267552	T	C	Weight	Neale B	0.02814	0.004039	3.241E-12	336227	IVNT

Alcohol use disorder	rs13107325	4	102267552	T	C	Body mass index	Speliotes EK	NA	NA	2E-13	-	-
Alcohol use disorder	rs7117878	11	57653985	A	C	Types of physical activity in last 4 weeks: heavy diy	Neale B	-0.007368	0.001268	6.147E-09	335599	risk diff
Alcohol use disorder	rs9926289	16	53786591	A	G	Body mass index	GIANT	0.07703	0.01338	8.551E-09	13991	IVNT
Alcohol use disorder	rs9926289	16	53786591	A	G	Body mass index	GIANT	0.0725	0.0052	6.44E-45	97745	IVNT
Alcohol use disorder	rs9926289	16	53786591	A	G	Hip circumference	GIANT	0.064	0.0057	5.5E-29	74367	IVNT
Alcohol use disorder	rs9926289	16	53786591	A	G	Waist circumference	GIANT	0.066	0.0054	4.5E-34	83784	IVNT
Alcohol use disorder	rs9926289	16	53786591	A	G	Waist hip ratio	GIANT	0.037	0.0053	6.2E-12	73257	IVNT
Alcohol use disorder	rs9926289	16	53786591	A	G	Body mass index	Neale B	0.07006	0.002457	1.186E-178	336107	IVNT
Alcohol use disorder	rs9926289	16	53786591	A	G	Hip circumference	Neale B	0.05899	0.00246	6.197E-127	336601	IVNT
Alcohol use disorder	rs9926289	16	53786591	A	G	Waist circumference	Neale B	0.05437	0.0022	1.108E-134	336639	IVNT
Alcohol use disorder	rs9926289	16	53786591	A	G	Weight	Neale B	0.05811	0.00217	1.04E-157	336227	IVNT
Problematic alcohol use	rs6421482	1	65954222	A	G	Body mass index	Neale B	-0.01376	0.002429	1.484E-08	336107	IVNT
Problematic alcohol use	rs6421482	1	65954222	A	G	Hip circumference	Neale B	-0.0139	0.002431	1.098E-08	336601	IVNT
Problematic alcohol use	rs6421482	1	65954222	A	G	Weight	Neale B	-0.01293	0.002145	1.667E-09	336227	IVNT
Problematic Alcohol use	rs1260326	2	27508073	C	T	Inflammatory bowel disease	IBDGC	-0.07711	0.009964	1E-14	-	log OR
Problematic Alcohol use	rs1260326	2	27508073	C	T	Weight	Neale B	0.01778	0.00217	2.53E-16	336227	IVNT
Problematic alcohol use	rs494904	2	44914041	C	T	Ever smoked	Neale B	0.006818	0.001221	2.368E-08	336067	risk diff
Problematic alcohol use	rs494904	2	44914041	C	T	Past tobacco smoking	Neale B	-0.0187	0.003254	9.124E-09	310749	-
Problematic alcohol use	rs62250713	3	85464643	A	G	Body mass index	Neale B	0.01711	0.002501	7.786E-12	336107	IVNT
Problematic alcohol use	rs62250713	3	85464643	A	G	Ever smoked	Neale B	0.008865	0.001238	7.901E-13	336067	risk diff
Problematic alcohol use	rs62250713	3	85464643	A	G	Past tobacco smoking	Neale B	-0.02635	0.003296	1.315E-15	310749	-
Problematic alcohol use	rs62250713	3	85464643	A	G	Smoking status: previous	Neale B	0.008495	0.001211	2.273E-12	336024	risk diff
Problematic alcohol use	rs62250713	3	85464643	A	G	Waist circumference	Neale B	0.01252	0.002239	2.277E-08	336639	IVNT
Problematic alcohol use	rs62250713	3	85464643	A	G	Weight	Neale B	0.0162	0.002209	2.3E-13	336227	IVNT
Problematic Alcohol use	rs13135092	4	102276925	A	G	Crohns disease	IBDGC	-0.2215	0.0389	1.21E-08	20883	log OR
Problematic alcohol use	rs13135092	4	102276925	A	G	Body mass index	Neale B	-0.05005	0.004374	2.596E-30	336107	IVNT
Problematic alcohol use	rs13135092	4	102276925	A	G	Hip circumference	Neale B	-0.04007	0.00438	5.809E-20	336601	IVNT
Problematic alcohol use	rs13135092	4	102276925	A	G	Qualifications: college or university degree	Neale B	0.01152	0.002075	2.82E-08	334070	risk diff
Problematic alcohol use	rs13135092	4	102276925	A	G	Waist circumference	Neale B	-0.02425	0.003918	6.013E-10	336639	IVNT
Problematic alcohol use	rs13135092	4	102276925	A	G	Weight	Neale B	-0.02841	0.003864	1.955E-13	336227	IVNT
Problematic alcohol use	rs9937709	16	53786901	A	G	Body mass index	Neale B	-0.0662	0.002438	3.422E-162	336107	IVNT
Problematic alcohol use	rs9937709	16	53786901	A	G	Hip circumference	Neale B	-0.05597	0.002441	3.234E-116	336601	IVNT

Problematic alcohol use	rs9937709	16	53786901	A	G	Waist circumference	Neale B	-0.05194	0.002183	5.686E-125	336639	IVNT
Problematic alcohol use	rs9937709	16	53786901	A	G	Weight	Neale B	-0.05521	0.002154	9.411E-145	336227	IVNT
Problematic alcohol use	rs492602	19	48703160	G	A	Crohns disease	IBDGC	NA	NA	2.1E-09	14342	log OR
Problematic alcohol use	rs492602	19	48703160	G	A	Crohns disease	IBDGC	0.1353	0.0237	1.212E-08	20883	log OR

Table S7. Alcohol consumption-related CpG sites and mQTLs from ARIES database.

CpG	CpG.Chr	CpG.Pos	EWAS.beta	EWAS.SE	mQTL	mQTL.Chr	mQTL.Pos	Effect Allele	Other allele	MAF	ARIES.beta	ARIES.SE	Cis
cg00546117	1	8445545	-0.000208	3.86E-05	rs2708630	1	8447404	C	T	0.33	-0.74489	0.0268557	Y
cg07180646	1	15481541	-0.000229	3.90E-05	rs941456	1	15471731	T	C	0.053	-0.7429	0.08248286	Y
cg04605617	1	20501558	-0.00026	4.77E-05	rs10916683	1	20300544	G	A	0.163	-0.40198	0.05226425	Y
cg04605617	1	20501558	-0.00026	4.77E-05	rs2872909	1	20482701	A	G	0.187	-0.56744	0.04640759	Y
cg03260624	1	91970722	-0.000242	3.74E-05	rs13447450	1	91965850	T	C	0.368	0.22058	0.02819487	Y
cg16246545	1	120255941	-0.000606	8.57E-05	rs11583993	1	120255370	A	G	0.092	-0.75896	0.07416818	Y
cg19238380	1	156093948	-0.000295	4.20E-05	rs517606	1	156089324	T	G	0.043	-0.86261	0.10918727	Y
cg00313914	1	201618901	-0.000447	8.38E-05	rs34569491	1	201501963	T	C	0.429	0.44233	0.04309944	Y
cg00313914	1	201618901	-0.000447	8.38E-05	rs7364578	1	201616627	A	G	0.267	-0.59581	0.0471075	Y
cg23712594	1	226591890	-0.000197	3.29E-05	rs1433574	1	226609339	C	A	0.18	0.29472	0.03910761	Y
cg10456541	2	8721512	-0.00019	3.43E-05	rs59996641	2	8723959	T	A	0.271	-0.26204	0.03735547	Y
cg14274621	2	8825107	-0.000137	2.37E-05	rs11894184	2	8825011	C	T	0.069	0.86828	0.07161226	Y
cg00883689	2	54802904	-0.000285	4.09E-05	rs4455200	2	54801591	A	T	0.258	0.6648	0.04324282	Y
cg20945261	3	13460618	-0.000192	3.20E-05	rs11924012	3	13484654	C	G	0.128	0.28742	0.04154741	Y
cg24642820	3	13462465	-0.000302	5.27E-05	rs7628923	3	13439005	G	A	0.122	-0.44724	0.05376747	Y
cg18483487	3	45208900	-0.000206	3.69E-05	rs1010138	3	45220250	A	T	0.059	1.08612	0.08393762	Y
cg18483487	3	45208900	-0.000206	3.69E-05	rs62244501	3	45216595	T	C	0.177	-0.54528	0.05174176	Y
cg18482892	3	56833426	-0.000226	3.95E-05	rs35983891	3	56831267	T	C	0.412	-0.26371	0.03523302	Y
cg02578470	3	127320670	-0.000238	3.96E-05	rs2720262	3	127315788	A	C	0.423	-0.30423	0.03584765	Y
cg02282631	5	42953543	-0.000325	5.87E-05	rs36122053	5	42954404	A	C	0.129	-0.43384	0.06936645	Y
cg12807764	5	146864669	-0.000257	4.39E-05	rs7734086	5	146855963	T	C	0.488	0.36611	0.03283574	Y
cg12662084	6	17809126	-0.000349	5.44E-05	rs6509695	19	53339237	C	T	0.427	0.4727	0.03212545	N
cg17865955	6	33601177	-0.000211	3.42E-05	rs11760139	6	33587392	T	C	0.141	-0.81382	0.06134898	Y
cg17865955	6	33601177	-0.000211	3.42E-05	rs28360584	6	33606524	C	G	0.142	0.66489	0.05955661	Y
cg17865955	6	33601177	-0.000211	3.42E-05	rs6926568	6	33597079	T	C	0.255	-0.52372	0.04805339	Y
cg05593667	6	35490744	-0.000252	3.09E-05	rs7755718	6	35489971	G	A	0.054	-1.14105	0.07687131	Y
cg26312998	6	43337775	-0.000171	2.99E-05	rs70953670	6	43264895	A	C	0.11	1.0844	0.05783217	Y
cg17390562	6	159238463	-0.000141	2.33E-05	rs9346767	6	159197371	T	C	0.494	0.33971	0.03356553	Y

cg22871253	6	159238744	-0.000188	3.10E-05	rs9456354	6	159248864	C	G	0.499	0.295	0.03638593	Y
cg07512517	7	38408106	-0.000171	3.07E-05	rs12532845	7	38384841	A	G	0.241	-0.34803	0.04534392	Y
cg08597832	8	144416327	-0.000162	2.48E-05	rs2467943	8	144420030	C	T	0.092	0.61815	0.06950277	Y
cg23629150	8	144416404	-0.000241	4.27E-05	rs2467933	8	144428185	G	C	0.092	0.86949	0.05679404	Y
cg12188860	8	144416485	-0.000239	4.12E-05	rs2467936	8	144445222	A	G	0.1	0.99708	0.05643899	Y
cg12188860	8	144416485	-0.000239	4.12E-05	rs34148379	8	144390502	G	A	0.466	0.42516	0.03845267	Y
cg07104958	10	46168551	-0.000263	4.51E-05	rs4418752	10	46102971	C	T	0.034	1.20889	0.10972493	Y
cg23444264	10	72219751	-0.000193	3.38E-05	rs1106887	10	72219918	T	C	0.378	0.26452	0.04277601	Y
cg19939077	10	81108060	-0.000226	3.38E-05	rs2181080	10	81121960	G	A	0.463	-0.39695	0.04253767	Y
cg07091481	10	82169149	-0.000194	3.16E-05	rs6585994	10	82191643	A	G	0.482	0.219	0.03287311	Y
cg03599037	10	82172508	-0.000136	1.88E-05	rs72819578	10	82225610	T	C	0.042	0.65418	0.09730029	Y
cg00291478	10	121301041	-0.000339	5.85E-05	rs10787977	10	121298412	T	C	0.135	-0.39786	0.05003131	Y
cg20204986	11	32448067	-0.000178	3.31E-05	rs11824258	11	32442275	G	A	0.228	0.28932	0.0495338	Y
cg12616487	11	62379063	-0.000168	2.51E-05	rs12808829	11	62378660	A	G	0.349	-0.26998	0.0384346	Y
cg10816169	11	66080868	-0.000273	4.46E-05	rs3741368	11	66083782	A	G	0.47	0.31731	0.0434482	Y
cg03575969	11	72492171	-0.00017	3.16E-05	rs481206	11	72497462	T	C	0.344	-0.27029	0.04089757	Y
cg08115371	11	79115706	-0.000394	6.52E-05	rs7117144	11	78861603	C	T	0.047	-0.69348	0.10011304	Y
cg10045354	11	111169427	-0.000219	4.00E-05	rs11213823	11	111169990	T	C	0.287	-0.25406	0.04496804	Y
cg20374917	11	128603874	-0.000163	2.45E-05	rs607703	11	128604174	T	C	0.482	-0.30737	0.03357885	Y
cg27479634	12	54070545	-0.000217	3.67E-05	rs4759278	12	54055801	C	T	0.307	-0.51257	0.04552172	Y
cg24420742	12	77721808	-0.000197	3.43E-05	rs59836592	12	77671731	C	G	0.027	-1.42902	0.12374171	Y
cg18954900	12	96604497	-0.000234	3.87E-05	rs12299943	12	96611648	G	A	0.305	0.3219	0.04351735	Y
cg01687189	12	102225365	-0.000253	4.10E-05	rs4237981	12	102225611	A	T	0.49	-0.55921	0.0349073	Y
cg23975840	12	117042895	0.0002678	4.21E-05	rs7960700	12	117039310	G	A	0.19	0.36936	0.03830133	Y
cg00986580	14	22951241	-0.000245	4.14E-05	rs227860	14	22950491	A	T	0.13	0.72755	0.05858494	Y
cg03345232	14	92981121	-0.000209	3.38E-05	rs12884739	14	93019511	A	G	0.36	-0.47758	0.04588934	Y
cg03345232	14	92981121	-0.000209	3.38E-05	rs77826962	14	93006567	G	A	0.16	0.47557	0.06065487	Y
cg18125510	14	100841768	-0.000342	5.53E-05	rs12891492	14	100843343	G	A	0.228	-0.47942	0.04112782	Y
cg26430287	15	67439567	-0.000247	3.87E-05	rs56118922	15	67407252	G	A	0.122	-0.42369	0.05008855	Y
cg26430287	15	67439567	-0.000247	3.87E-05	rs62006055	15	67439629	G	A	0.272	0.48045	0.0340427	Y
cg07286682	15	69447132	0.0001971	3.53E-05	rs62008108	15	69440889	C	A	0.42	0.36686	0.04286339	Y
cg03603530	16	69365009	-0.000109	1.93E-05	rs1127231	16	69354963	G	A	0.312	0.57129	0.04416936	Y

cg14330293	17	1374051	0.0001286	2.25E-05	rs4411554	17	1319765	C	G	0.296	-0.23463	0.04053102	Y
cg05465916	17	7819762	-0.000191	3.19E-05	rs138596240	17	7923609	G	A	0.018	-1.18004	0.16394955	Y
cg01735398	17	18907305	-0.000228	4.25E-05	rs73306439	17	18866861	T	G	0.039	0.87878	0.11468506	Y
cg22332722	18	25754857	-0.000361	6.46E-05	rs1944288	18	25749095	C	A	0.246	-0.33898	0.04744217	Y
cg16705273	18	42262885	-0.000251	3.47E-05	rs12456054	18	42077425	A	G	0.127	-0.457	0.06904304	Y
cg16705273	18	42262885	-0.000251	3.47E-05	rs78646871	18	42262428	G	A	0.025	-0.93993	0.1355022	Y
cg15059065	19	17354961	-0.000243	4.00E-05	rs74182298	19	17346953	G	A	0.3	-0.487	0.04651967	Y
cg15059065	19	17354961	-0.000243	4.00E-05	rs891202	19	17338033	T	C	0.378	0.34603	0.04842574	Y
cg22052056	20	31351813	-0.00018	3.25E-05	rs34467919	20	31272928	T	C	0.388	-0.24386	0.03178505	Y
cg14391586	20	62681296	-0.000188	3.51E-05	rs816943	20	62676009	G	A	0.48	0.57082	0.03924937	Y
cg00549412	21	43933482	-0.000191	3.40E-05	rs6586324	21	43942135	T	C	0.49	0.33801	0.04040833	Y

Table S8. Two-sample Mendelian Randomization estimates of methylation at alcohol consumption-related CpG sites on CRC risk.

CpG site	Chr	Position	Nearest Gene(s)	Method	SNP	OR	LCI	UCI	P Value
cg00546117	1	8445545	*RERE*	Wald Ratio	1	1.02	0.98	1.07	0.238
cg07180646	1	15481541	*TMEM51*	Wald Ratio	1	0.99	0.91	1.07	0.718
cg04605617	1	20501558	*PLA2G2C*	IVW	2	0.99	0.93	1.04	0.635
cg03260624	1	91970722	*CDC7*	Wald Ratio	1	1.04	0.91	1.19	0.549
cg16246545	1	120255941	*PHGDH*	Wald Ratio	1	1.06	0.99	1.13	0.08
cg19238380	1	156093948	*LMNA*	Wald Ratio	1	1.02	0.94	1.1	0.715
cg00313914	1	201618901	*NAV1*	IVW	2	0.99	0.95	1.04	0.762
cg23712594	1	226591890	*PARP1*	Wald Ratio	1	0.9	0.8	1.03	0.124
cg14274621	2	8825107	-	Wald Ratio	1	1	0.93	1.07	0.938
cg00883689	2	54802904	*SPTBN1*	Wald Ratio	1	1.02	0.97	1.08	0.351
cg20945261	3	13460618	*NUP210*	Wald Ratio	1	0.97	0.84	1.12	0.684
cg24642820	3	13462465	*NUP210*	Wald Ratio	1	0.97	0.88	1.07	0.595
cg18483487	3	45208900	-	IVW	2	1.02	0.97	1.06	0.479
cg18482892	3	56833426	*ARHGEF3*	Wald Ratio	1	0.92	0.82	1.03	0.141
cg02578470	3	127320670	*MCM2*	Wald Ratio	1	0.98	0.89	1.08	0.659
cg02282631	5	42953543	-	Wald Ratio	1	0.96	0.87	1.06	0.467
cg12807764	5	146864669	*DPYSL3*	Wald Ratio	1	1.02	0.94	1.1	0.65
cg17865955	6	33601177	*ITPR3*	IVW	3	1.02	0.98	1.05	0.301
cg07512517	7	38408106	*TRG-AS1, TRGV1*	Wald Ratio	1	0.94	0.86	1.04	0.243
cg08597832	8	144416327	*TOP1MT*	Wald Ratio	1	1	0.92	1.09	0.974
cg23629150	8	144416404	*TOP1MT*	Wald Ratio	1	1	0.94	1.07	0.965
cg12188860	8	144416485	*TOP1MT*	IVW	2	1.02	0.97	1.07	0.41
cg07104958	10	46168551	*ZFAND4*	Wald Ratio	1	0.99	0.92	1.05	0.652
cg23444264	10	72219751	-	Wald Ratio	1	1.11	0.93	1.34	0.249
cg19939077	10	81108060	*PPIF*	Wald Ratio	1	0.93	0.87	1	0.053
cg07091481	10	82169149	*FAM213A*	Wald Ratio	1	0.95	0.83	1.08	0.451
cg03599037	10	82172508	*FAM213A*	Wald Ratio	1	0.98	0.87	1.11	0.782
cg00291478	10	121301041	*RGS10*	Wald Ratio	1	1.04	0.94	1.16	0.448

cg20204986	11	32448067	*WT1*	Wald Ratio	1	1.05	0.93	1.18	0.405
cg12616487	11	62379063	*ROM1, EML3*	Wald Ratio	1	1.06	0.95	1.18	0.322
cg10816169	11	66080868	*RP11-867G23.13*	Wald Ratio	1	1.03	0.94	1.13	0.513
cg08115371	11	79115706	*ODZ4*	Wald Ratio	1	0.95	0.87	1.05	0.333
cg20374917	11	128603874	*FLI1*	Wald Ratio	1	1.05	0.95	1.15	0.354
cg27479634	12	54070545	*ATP5G2*	Wald Ratio	1	0.97	0.91	1.04	0.378
cg24420742	12	77721808	*NAV3*	Wald Ratio	1	0.98	0.92	1.04	0.476
cg18954900	12	96604497	*ELK3*	Wald Ratio	1	0.95	0.87	1.05	0.345
cg01687189	12	102225365	*GNPTAB*	Wald Ratio	1	0.98	0.93	1.03	0.404
cg23975840	12	117042895	-	Wald Ratio	1	0.93	0.84	1.03	0.164
cg00986580	14	22951241	*TRAJ54, TRAJ53, AE000661.37*	Wald Ratio	1	0.93	0.87	1	0.067
cg03345232	14	92981121	*RIN3*	IVW	2	0.97	0.92	1.02	0.255
cg18125510	14	100841768	*WARS, WDR25*	Wald Ratio	1	1.03	0.96	1.11	0.354
cg26430287	15	67439567	*SMAD3, RP11-342M21.2*	IVW	2	0.98	0.93	1.05	0.617
cg07286682	15	69447132	-	Wald Ratio	1	0.99	0.91	1.07	0.746
cg03603530	16	69365009	*RP11-343C2.12, PDF, COG8*	Wald Ratio	1	1.03	0.97	1.08	0.362
cg14330293	17	1374051	*MYO1C*	Wald Ratio	1	0.97	0.85	1.11	0.668
cg05465916	17	7819762	*AC025335.1*	Wald Ratio	1	1.02	0.93	1.11	0.672
cg01735398	17	18907305	*SLC5A10*	Wald Ratio	1	1.07	0.93	1.24	0.339
cg22332722	18	25754857	*CDH2*	Wald Ratio	1	1.04	0.94	1.15	0.406
cg16705273	18	42262885	*SETBP1*	IVW	2	0.97	0.9	1.04	0.393
cg15059065	19	17354961	*NR2F6, AC010646.3*	IVW	2	0.99	0.94	1.05	0.831
cg22052056	20	31351813	*DNMT3B*	Wald Ratio	1	1.02	0.9	1.15	0.733
cg14391586	20	62681296	*SOX18*	Wald Ratio	1	0.99	0.95	1.05	0.826
cg00549412	21	43933482	*SLC37A1*	Wald Ratio	1	0.99	0.91	1.08	0.829

Table S9. Characteristics of CRC incident cases and population-based controls nested in the UK Biobank.

Characteristics		Cases	Controls	Overall
**Age at recruitment, mean (SD)**		61.7 (6.2)	61.8 (6.3)	61.8 (6.3)
**Gender, n (%)**	Male	702 (37.1)	3671 (39.1)	4373 (38.8)
	Female	1190 (62.9)	5715 (60.9)	6905 (61.2)
**BMI, n (%)**	Normal weight	519 (27.4)	2836 (30.2)	3355 (29.7)
	Overweight	886 (46.8)	4423 (47.1)	5309 (47.1)
	Obese	472 (24.9)	2075 (22.1)	2547 (22.6)
	Underweight	5 (0.3)	26 (0.3)	31 (0.3)
	NA	10 (0.5)	26 (0.3)	36 (0.3)
**Smoking status, n (%)**	Current smoker	174 (9.2)	823 (8.8)	997 (8.8)
	Previous smoker	888 (46.9)	3811 (40.6)	4699 (41.7)
	Never smoker	823 (43.5)	4718 (50.3)	5541 (49.1)
	NA	7 (0.4)	34 (0.4)	41 (0.4)
**Aspirin use, n (%)**	User	359 (19.0)	1775 (18.9)	2134 (18.9)
	Non-user	1512 (79.9)	7508 (80.0)	9020 (80.0)
	NA	21 (1.1)	103 (1.1)	124 (1.1)
**Drinking status, n (%)**	Never drinker	81 (4.3)	456 (4.9)	537 (4.8)
	Light drinker	491 (26.0)	2720 (29.0)	3211 (28.5)
	Moderate drinker	998 (52.7)	5119 (54.5)	6117 (54.2)
	Heavy drinker	322 (17.0)	1091 (11.6)	1413 (12.5)
**Genotype of rs3087967, n (%)**	CC	193 (10.2)	876 (9.3)	1069 (9.5)
	CT	838 (44.3)	4030 (42.9)	4868 (43.2)
	TT	861 (45.5)	4480 (47.7)	5341 (47.4)

Supplementary figures

Figure S1. Flowchart of the selection of eligible studies for meta-analysis.

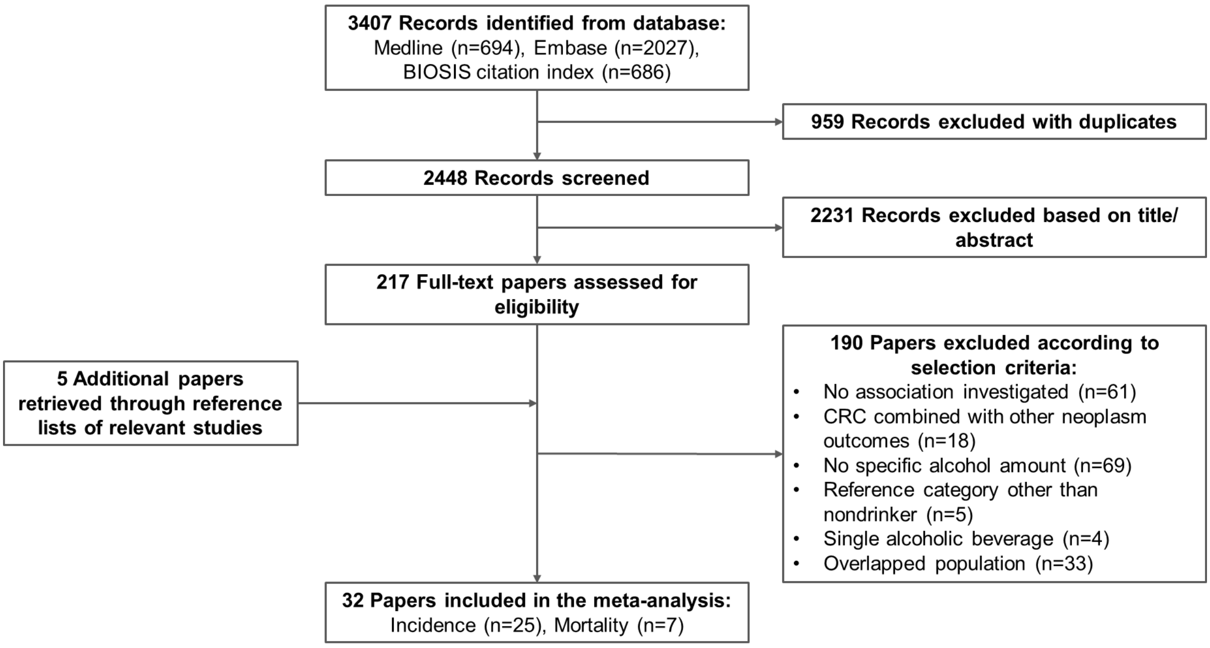

CRC, colorectal cancer.

Figure S2. Pooled RR of CRC risk for light, moderate and heavy drinkers in the meta-analysis. Light, <12.5 g/d; Moderate, 12.5-50 g/d; Heavy, >50 g/d.

Group	Events	Studies	Exposure	RR	95% CI	P value	I^2(%)
**Incidence**	237333	23	Light	1.02	[0.99, 1.06]	0.234	31.60
		25	Moderate	1.24	[1.17, 1.32]	5.27×10^{-12}	54.90
		5	Heavy	1.54	[1.15, 2.06]	0.004	74.60
**Mortality**	3659	7	Light	0.98	[0.88, 1.10]	0.789	0.00
		7	Moderate	0.98	[0.86, 1.12]	0.789	0.00
		5	Heavy	1.16	[0.85, 1.57]	0.359	9.70
**Men**	>30214	19	Light	1.04	[0.99, 1.09]	0.104	19.20
		20	Moderate	1.24	[1.15, 1.33]	7.26×10^{-9}	50.90
		7	Heavy	1.45	[1.16, 1.80]	0.001	75.80
**Women**	>43924	15	Light	0.97	[0.92, 1.02]	0.215	40.80
		17	Moderate	1.06	[1.00, 1.12]	0.061	18.40
		3	Heavy	1.05	[0.78, 1.41]	0.748	70.80
**Colon**	27647	13	Light	0.99	[0.93, 1.06]	0.825	27.60
		14	Moderate	1.25	[1.14, 1.38]	5.45×10^{-6}	58.80
		4	Heavy	1.59	[1.06, 2.38]	0.026	80.70
**Rectum**	12042	9	Light	1.08	[0.99, 1.18]	0.094	25.70
		9	Moderate	1.38	[1.28, 1.48]	1.65×10^{-16}	1.30
		4	Heavy	1.92	[1.47, 2.51]	2.17×10^{-6}	27.60
**Proximal colon**	>10822	4	Light	0.94	[0.88, 1.00]	0.063	1.00
		5	Moderate	1.10	[0.97, 1.25]	0.157	63.80
		1	Heavy	1.35	[0.97, 1.89]	0.078	–
**Distal colon**	>8656	4	Light	1.04	[0.90, 1.19]	0.618	60.00
		5	Moderate	1.24	[1.15, 1.35]	1.25×10^{-7}	0.00
		1	Heavy	1.66	[1.17, 2.36]	0.005	–
**European population**	37219	12	Light	1.03	[0.96, 1.11]	0.361	50.60
		13	Moderate	1.20	[1.10, 1.31]	4.39×10^{-5}	48.70
		2	Heavy	1.41	[1.16, 1.71]	4.24×10^{-4}	0.00
**Asian population**	160627	10	Light	1.00	[0.89, 1.11]	0.952	0.00
		11	Moderate	1.29	[1.09, 1.54]	0.003	53.60
		6	Heavy	1.53	[1.02, 2.29]	0.038	61.50

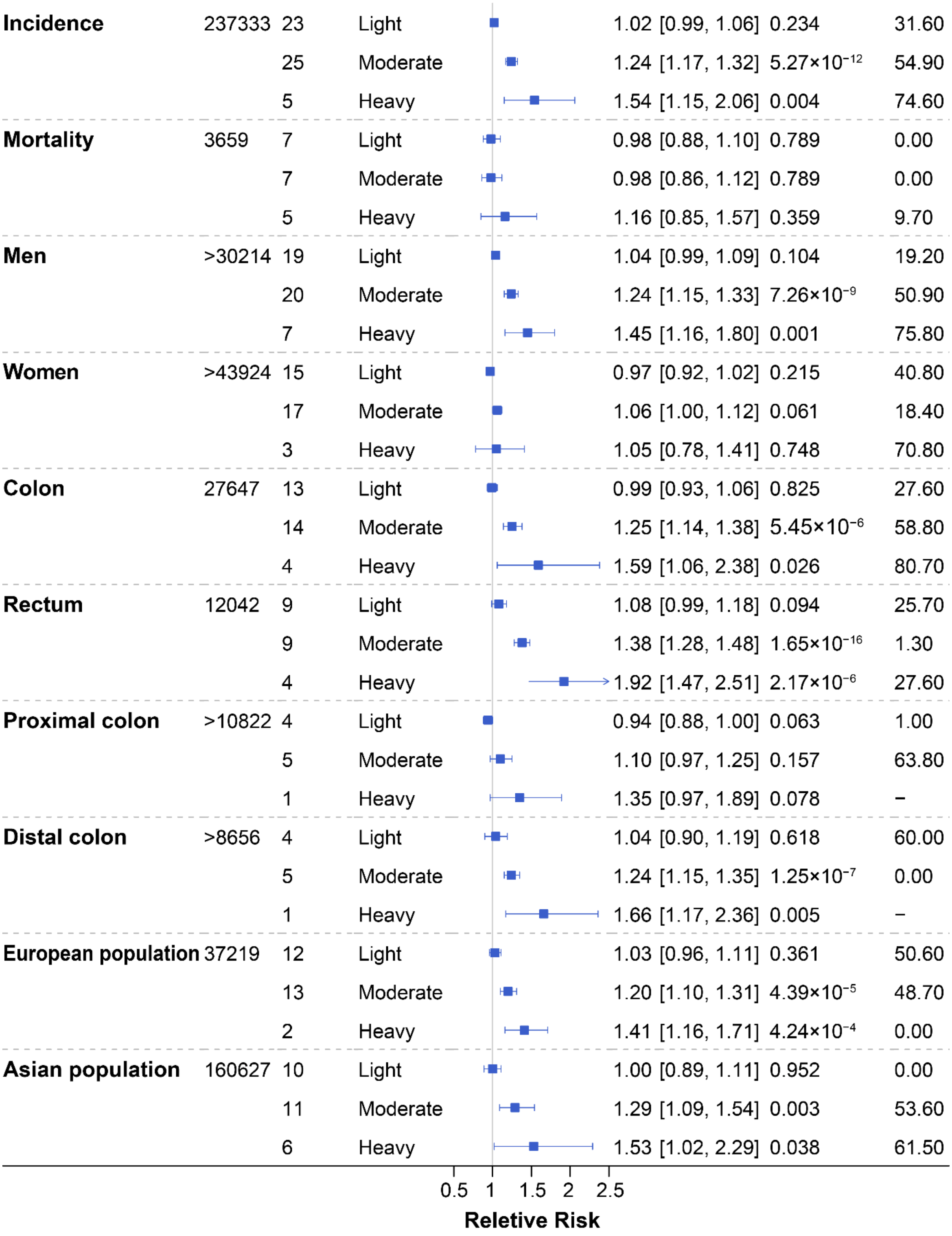

Figure S3. Dose-response effect of alcohol drinking on CRC risk.

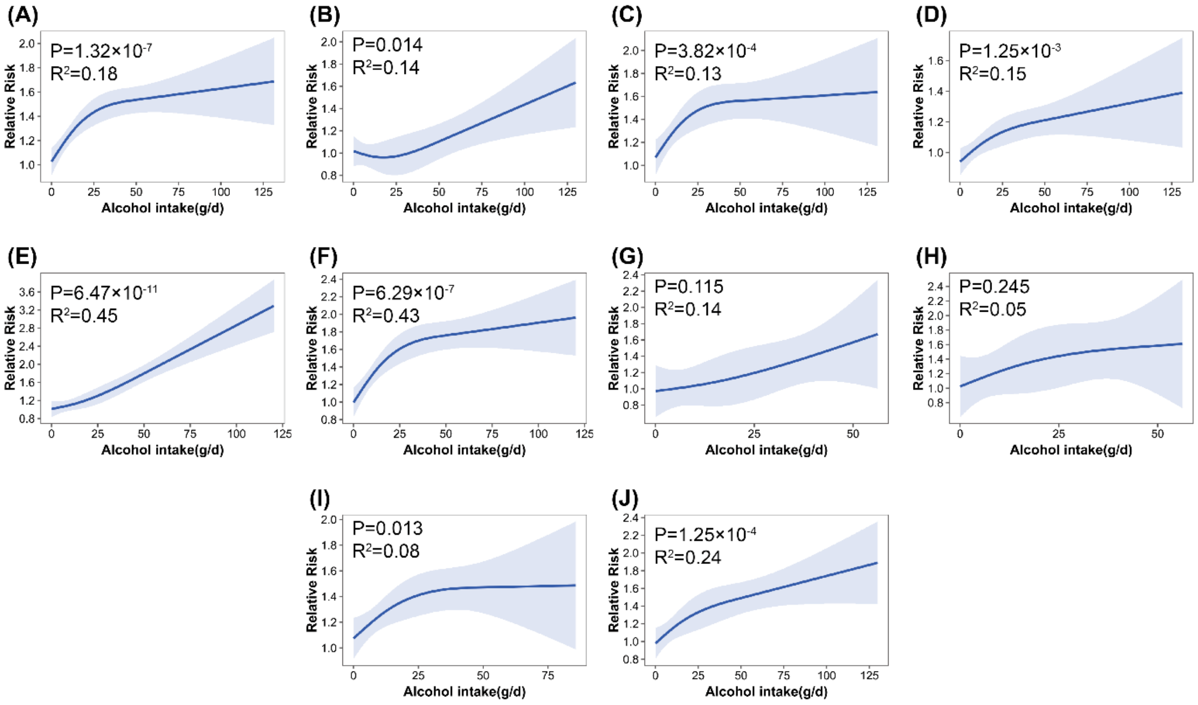

R^2, adjusted R-square of the cubic-spline model. (A) Risk of CRC incidence; (B) Risk of CRC mortality; (C) Risk of men drinkers; (D) Risk of women drinkers; (E) Risk of rectum cancer; (F) Risk of colon cancer; (G) Risk of proximal colon cancer; (H) Risk of distal colon cancer; (I) Risk of European mainly population; (J) Risk of Asian mainly population.

Figure S4. Regional plots of cg10045354 and mapped genes.

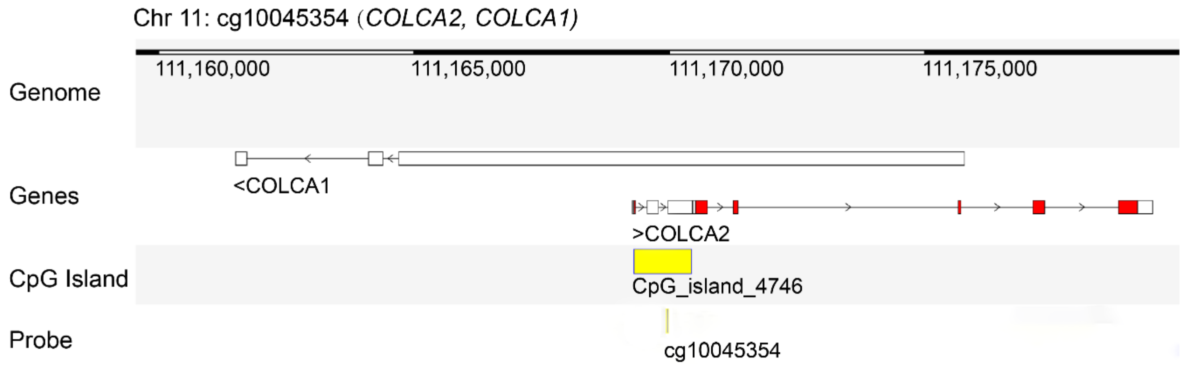

Figure S5. Single-tissue eQTL plot of rs11213823 (cg10045354, COLCA2).

Tissue	Samples	NES	p-value	m-value
Colon - Transverse	368	0.570	3.9e-37	1.00
Cells - EBV-transformed lymphocytes	147	0.427	2.8e-5	1.00
Small Intestine - Terminal Ileum	174	0.349	4.1e-8	1.00
Brain - Spinal cord (cervical c-1)	126	0.340	1.1e-3	0.992
Spleen	227	0.275	2.9e-4	1.00
Prostate	221	0.242	5.5e-5	1.00
Nerve - Tibial	532	0.219	1.4e-8	1.00
Colon - Sigmoid	318	0.211	1.9e-5	1.00
Uterus	129	0.210	0.06	0.828
Brain - Hippocampus	165	0.200	3.8e-3	1.00
Artery - Tibial	584	0.196	3.9e-6	1.00
Artery - Aorta	387	0.188	1.4e-5	1.00
Brain - Substantia nigra	114	0.183	0.04	0.724
Artery - Coronary	213	0.173	4.1e-3	0.985
Brain - Cerebellum	209	0.172	4.0e-3	0.987
Brain - Anterior cingulate cortex (BA24)	147	0.169	0.005	0.939
Esophagus - Mucosa	497	0.154	3.3e-5	0.998
Brain - Caudate (basal ganglia)	194	0.130	0.009	0.838
Lung	515	0.118	5.8e-4	0.603
Brain - Frontal Cortex (BA9)	175	0.114	0.04	0.541
Esophagus - Gastroesophageal Junction	330	0.105	0.05	0.378
Breast - Mammary Tissue	396	0.0979	0.009	0.178
Brain - Cerebellar Hemisphere	175	0.0948	0.2	0.170
Pancreas	305	0.0895	0.06	0.0990
Brain - Nucleus accumbens (basal ganglia)	202	0.0886	0.09	0.0960
Ovary	167	0.0875	0.2	0.247
Testis	322	0.0867	0.08	0.164
Brain - Cortex	205	0.0833	0.08	0.0500
Esophagus - Muscularis	465	0.0813	0.03	0.0270
Brain - Amygdala	129	0.0786	0.3	0.193
Stomach	324	0.0779	0.07	0.0240
Brain - Putamen (basal ganglia)	170	0.0736	0.1	0.0470
Skin - Not Sun Exposed (Suprapubic)	517	0.0675	0.1	0.00100
Adrenal Gland	233	0.0659	0.3	0.0640
Brain - Hypothalamus	170	0.0578	0.4	0.0690
Muscle - Skeletal	706	0.0400	0.3	0.00
Skin - Sun Exposed (Lower leg)	605	0.0316	0.3	0.00
Adipose - Subcutaneous	581	0.0304	0.4	0.00
Adipose - Visceral (Omentum)	469	0.0302	0.4	0.00
Whole Blood	670	-	-	-
Kidney - Cortex	73	-0.00807	1	0.142
Thyroid	574	-0.0177	0.5	0.00
Pituitary	237	-0.0343	0.6	0.00
Cells - Cultured fibroblasts	483	-0.0414	0.3	0.00
Heart - Left Ventricle	386	-0.0604	0.2	0.00
Minor Salivary Gland	144	-0.0762	0.2	0.00
Vagina	141	-0.0933	0.3	0.00
Heart - Atrial Appendage	372	-0.114	0.07	0.00
Liver	208	-0.195	0.01	0.00

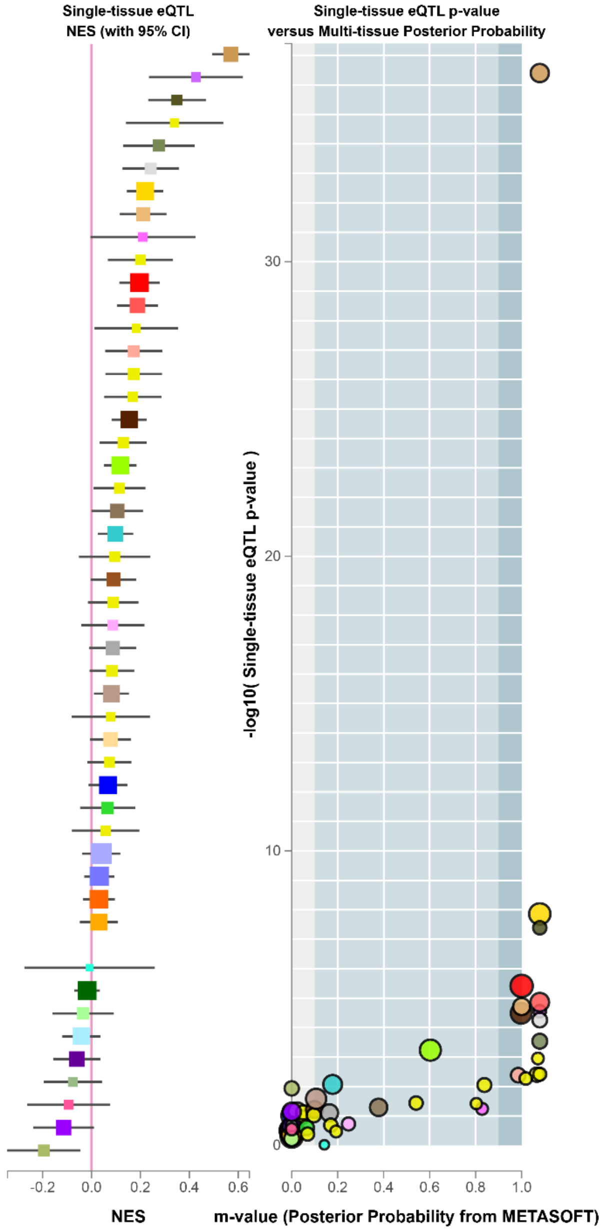

NES, normalized effect size. m value, the posterior probability that an eQTL effect exists in each tissue tested in the cross-tissue meta-analysis. Small m value (e.g., <0.1), the tissue is predicted to NOT have an eQTL effect; large m value (e.g., >0.9), the tissue is predicted to Have an eQTL effect; otherwise, the prediction of the existence of an eQTL effect is ambiguous.

Figure S6. Single-tissue eQTL plot of rs11213823 (cg10045354, COLCA1).

Tissue	Samples	NES	p-value	m-value
Colon - Transverse	368	0.434	8.2e-22	0.00
Cells - EBV-transformed lymphocytes	147	0.395	1.8e-3	0.00
Spleen	227	0.306	3.5e-5	0.00
Small Intestine - Terminal Ileum	174	0.137	0.04	0.00
Brain - Nucleus accumbens (basal ganglia)	202	0.0604	0.3	0.00
Brain - Spinal cord (cervical c-1)	126	0.0333	0.8	0.324
Brain - Caudate (basal ganglia)	194	0.0261	0.7	0.0120
Brain - Substantia nigra	114	0.0243	0.8	0.295
Cells - Cultured fibroblasts	483	-	-	-
Whole Blood	670	-	-	-
Prostate	221	-0.00391	0.9	0.00
Lung	515	-0.0347	0.2	0.00
Stomach	324	-0.0408	0.2	0.00
Ovary	167	-0.0434	0.5	0.148
Brain - Putamen (basal ganglia)	170	-0.0494	0.5	0.410
Uterus	129	-0.0558	0.6	0.555
Brain - Frontal Cortex (BA9)	175	-0.0724	0.3	0.683
Colon - Sigmoid	318	-0.0890	0.04	0.630
Brain - Hippocampus	165	-0.0895	0.2	0.714
Adrenal Gland	233	-0.0990	0.08	0.816
Artery - Aorta	387	-0.101	2.7e-3	0.918
Brain - Cerebellum	209	-0.103	0.2	0.838
Breast - Mammary Tissue	396	-0.112	2.1e-3	0.991
Brain - Amygdala	129	-0.134	0.09	0.896
Thyroid	574	-0.139	9.9e-8	1.00
Artery - Tibial	584	-0.143	9.9e-5	1.00
Esophagus - Mucosa	497	-0.146	1.3e-4	1.00
Adipose - Visceral (Omentum)	469	-0.146	5.8e-5	1.00
Skin - Sun Exposed (Lower leg)	605	-0.156	3.7e-6	1.00
Artery - Coronary	213	-0.161	1.2e-3	0.992
Kidney - Cortex	73	-0.163	0.06	0.936
Minor Salivary Gland	144	-0.164	0.01	0.985
Brain - Hypothalamus	170	-0.169	0.02	0.963
Adipose - Subcutaneous	581	-0.171	1.2e-6	1.00
Brain - Cortex	205	-0.177	0.008	0.984
Skin - Not Sun Exposed (Suprapubic)	517	-0.180	5.1e-6	1.00
Muscle - Skeletal	706	-0.192	4.4e-6	1.00
Brain - Cerebellar Hemisphere	175	-0.193	0.05	0.966
Pancreas	305	-0.195	4.5e-4	1.00
Brain - Anterior cingulate cortex (BA24)	147	-0.197	0.01	1.00
Esophagus - Gastroesophageal Junction	330	-0.209	8.7e-5	1.00
Heart - Atrial Appendage	372	-0.227	7.6e-5	1.00
Vagina	141	-0.248	0.005	1.00
Esophagus - Muscularis	465	-0.266	2.2e-13	1.00
Heart - Left Ventricle	386	-0.270	3.1e-8	1.00
Pituitary	237	-0.283	3.2e-6	1.00
Testis	322	-0.299	4.8e-9	1.00
Nerve - Tibial	532	-0.344	6.2e-20	1.00
Liver	208	-0.440	6.7e-7	1.00

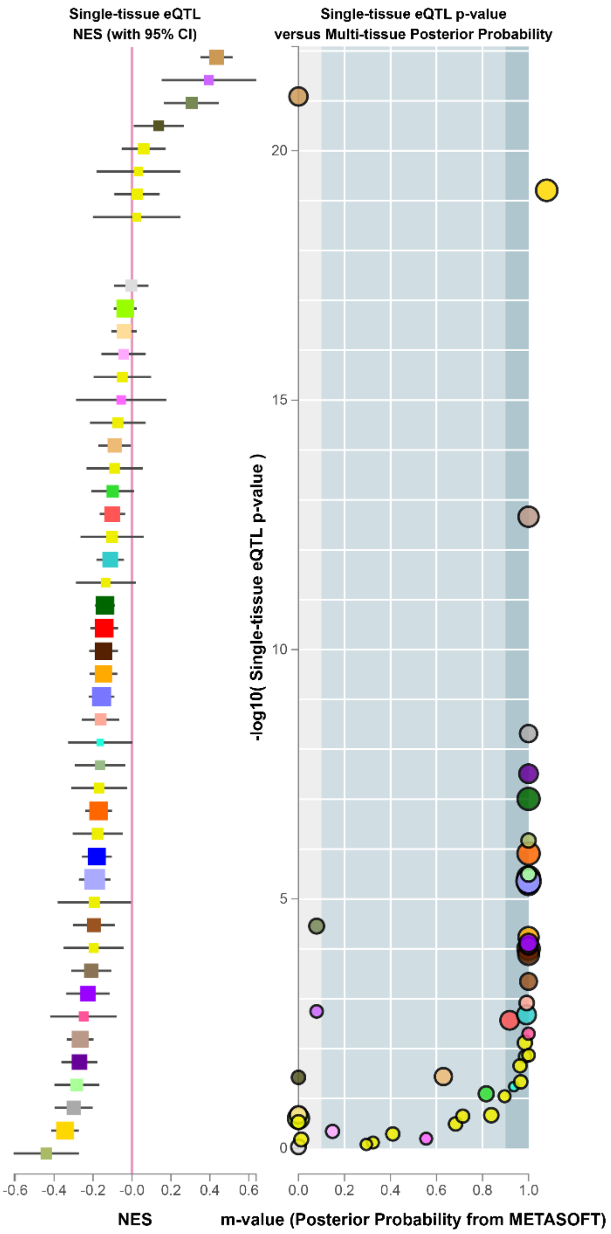

NES, normalized effect size. m value, the posterior probability that an eQTL effect exists in each tissue tested in the cross-tissue meta-analysis. Small m value (e.g., <0.1), the tissue is predicted to NOT have an eQTL effect; large m value (e.g., >0.9), the tissue is predicted to Have an eQTL effect; otherwise, the prediction of the existence of an eQTL effect is ambiguous.
